# Cholesterol-Lowering Treatment in Chronic Kidney Disease: Multistage Pairwise and Network Meta-Analyses

**DOI:** 10.1038/s41598-019-45431-5

**Published:** 2019-06-20

**Authors:** Francisco Herrera-Gómez, M. Montserrat Chimeno, Débora Martín-García, Frank Lizaraso-Soto, Álvaro Maurtua-Briseño-Meiggs, Jesús Grande-Villoria, Juan Bustamante-Munguira, Eric Alamartine, Miquel Vilardell, Carlos Ochoa-Sangrador, F. Javier Álvarez

**Affiliations:** 1 Pharmacological Big Data Laboratory, Pharmacology and Therapeutics, Faculty of Medicine, University of Valladolid, Valladolid, Spain; 2 Nephrology, Hospital Virgen de la Concha – Sanidad de Castilla y León, Zamora, Spain; 3 Internal Medicine, Hospital Virgen de la Concha – Sanidad de Castilla y León, Zamora, Spain; 4 Cardiovascular risk unit, Hospital Clínico Universitario de Valladolid – Sanidad de Castilla y León, Valladolid, Spain; 5 Instituto de Investigación de la Facultad de Medicina Humana, Universidad de San Martín de Porres, Lima, Peru; 6 Woodland Medical Practice – NHS, Lincolnshire, United Kingdom; 7 Cardiac Surgery, Hospital Clínico Universitario de Valladolid – Sanidad de Castilla y León, Valladolid, Spain; 8 Nephrology, Dialysis and Transplantation, Centre Hospitalier Universitaire de Saint-Etienne, Saint-Priest-en-Jarez, France; 9 Medicine, Faculty of Medicine, Universidad Autónoma de Barcelona, Barcelona, Spain; 10 Research unit, Hospital Virgen de la Concha – Sanidad de Castilla y León, Zamora, Spain; 11 CEIm Área de Salud Valladolid Este, Hospital Clínico Universitario de Valladolid, Valladolid, Spain

**Keywords:** Nephrology, Risk factors

## Abstract

Pairwise and network meta-analyses on the relationship between the efficacy of the use of statins with or without ezetimibe and reductions in low-density lipoprotein cholesterol (LDLc) and C-reactive protein (CRP) in patients with chronic kidney disease (CKD) are presented. In the pairwise meta-analysis, statins with or without ezetimibe were shown to be efficacious in reducing major adverse cardiovascular events (MACE) in patients with CKD and an estimated glomerular filtration rate (eGFR) of less than 60 ml/min/1.73 m^2^, in the context of both primary prevention [odds ratio (OR)/95% confidence interval (95% CI)/I^2^/number of studies (n): 0.50/0.40–0.64/0%/6] and primary/secondary prevention (0.66/0.57–0.76/57%/18). However, in the Bayesian network meta-analysis, compared to the placebo, only atorvastatin 80 mg daily and atorvastatin and rosuvastatin at doses equivalent to simvastatin 20 mg daily reduced the odds of MACEs in this patient population. The network meta-analysis for LDLc and CRP treatment objectives also showed that, regardless of eGFR and excluding dialysis patients, the number of MACEs decreased in patients with CKD, with reductions in both LDLc and CRP of less than 50% (surface under the cumulative ranking (SUCRA)/heterogeneity (vague)/n: 0.77/0.14/3). The evaluation of the benefits of drugs may lead to individualized therapy for CKD patients: Cholesterol-lowering treatment for CKD patients with high levels of both LDLc and CRP is suggested.

## Introduction

Cardiovascular disease (CVD) occurs early in individuals with chronic kidney disease (CKD)^1^: Inflammation is central to the development of the first and subsequent CVD events in CKD patients (as well as in non-CKD patients)^2^ and plays an essential role in linking CVD and kidney function decline^1^. However, classic predictors of coronary and cerebrovascular events seem to perform better in the early stages of the CKD spectrum^3^. Importantly, in CKD patients, potential new markers of CVD events that are derived from analyses of the complex pathophysiology of CKD act as independent prognostic factors^4,5^. Despite advances in the qualification of biomarkers for the use of medicines^6^, the translation of real treatment effect modifiers for CVD in the CKD population is needed to evaluate the effects of the available therapies^7^.

The risk of coronary events in patients with CKD is high^8^, and the related evidence is in favor of reducing CVD risk using cholesterol-lowering treatment strategies^9^. However, the relationship between serum levels of low-density lipoprotein cholesterol (LDLc) and CVD is more difficult to understand in the context of CKD; malnutrition and chronic inflammation are additional factors to consider as CKD progresses, and these factors increase the risk of death in CKD patients^9^.

### Current context and study objective

The Kidney Disease–Improving Global Outcomes (KDIGO) guidelines recommend the use of statins with or without ezetimibe for adults 50 years of age or older with CKD and an estimated glomerular filtration rate (eGFR) of lower than 60 ml/min/1.73 m^2^ (KDIGO GFR categories G3a to G5) who are not being treated with chronic dialysis or kidney transplantation and not aiming to achieve specific LDLc targets^10^. Statins are also recommended for individuals older than 50 years when the eGFR is 60 ml/min/1.73 m^2^ or higher (KDIGO GFR categories G1 to G2).

Notably, in the evaluation of the impact of cholesterol-lowering treatment along the wide spectrum of CKD, an unanswered question remains: are statins with or without ezetimibe similarly efficacious in patients with an eGFR lower than 60 ml/min/1.73 m^2^ and in patients with an eGFR of 60 ml/min/1.73 m^2^ or higher? If not, which cholesterol-lowering treatment strategies are the best for lowering CVD risk in patients with an eGFR lower than 60 ml/min/1.73 m^2^? Furthermore, is the cardiovascular efficacy of statins with or without ezetimibe related to reductions in LDLc and C-reactive protein (CRP) in CKD patients as is the case for LDLc in non-CKD patients? Conventional pairwise meta-analysis may be insufficient to answer these questions, as the efficacy between multiple interventions that compare cholesterol-lowering treatments and placebo/no treatment cannot be studied separately. Only one possibility exists based on this technique: to compare cholesterol-lowering treatments as a whole with placebo/no treatment. The same concept applies for the reduction in LDLc and CRP with respect to the sole reduction in LDLc when meta-analysis on exposures is performed. Network meta-analysis provides a viable solution even though some interventions/exposures have never been compared head-to-head in randomized controlled trials that evaluate the cardiovascular efficacy of statins with or without ezetimibe in the context of CKD.

These multistage pairwise and network meta-analyses presents (1) a summary of the efficacy of statins with or without ezetimibe along the wide spectrum of CKD and (2) the relation of this treatment to reductions in LDLc and CRP with the aim of evaluating the real impact of cholesterol lowering-treatment on CVD in CKD patients.

## Results

### Literature search

**Figure 1:**
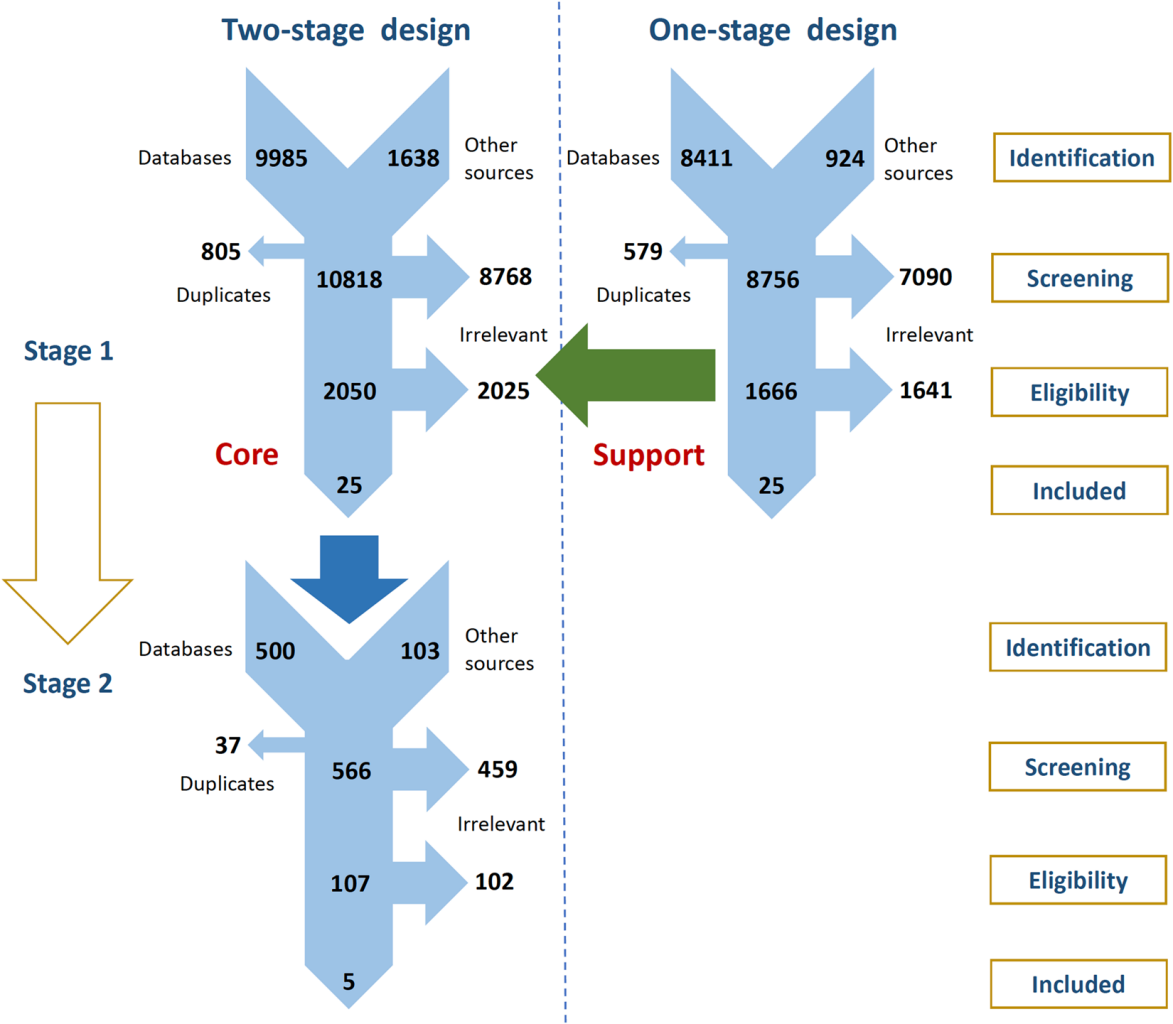
The PRISMA flowcharts presenting the selection processes used in the two systematic reviews. PRISMA, Preferred Reporting Items for Systematic Reviews and Meta-Analyses.

Data from 106 050 participants from 22 randomized controlled trials (RCT) are presented here^11–35^. Figure 1 presents the Preferred Reporting Items for Systematic reviews and Meta-Analyses (PRISMA) flowchart corresponding to the two systematic reviews^36^. Irrelevant citations were mostly interventional or observational studies of patients with early CKD (KDIGO GFR categories G1 to G2) that did not assess CVD-related outcomes or studies involving patients without CKD but affected by other pathologies. All of the following RCTs that were eligible at the treatment objectives evaluation phase had already been included at the cholesterol-lowering treatment efficacy evaluation phase: The Study of Heart and Renal Protection (SHARP)^17^, the Air Force/Texas Coronary Atherosclerosis Prevention study (AFCAPS/TexCAPS)^23,37^, the Collaborative Atorvastatin Diabetes Study (CARDS)^26,38^, and the Justification for the Use of Statins in Primary Prevention–an Intervention Trial Evaluating Rosuvastatin (JUPITER) study^28,39^.

All these pharmaceutical-industry-sponsored trials were published in peer-reviewed journals and corresponded to the phase 3 or 4 evaluation of various statins with or without ezetimibe, with both administered per os. Nineteen of these trials separately assessed persons with normal kidney function (NKF)/CKD KDIGO GFR categories G1–G2 and patients with CKD KDIGO GFR categories G3a–G5 but not patients treated with chronic dialysis^16–35^; 12 of the abovementioned studies were post hoc analyses of other RCTs conceived to evaluate participants from the general population or patients with other pathologies^22–33^. Five RCTs studied dialysis patients (hemodialysis and peritoneal dialysis modalities)^13–18^, and 1 RCT evaluated patients who underwent kidney transplantation^11,12^. The eligible RCTs are presented in Table S1.

### Cholesterol-lowering treatment efficacy evaluation

**Figure 2:**
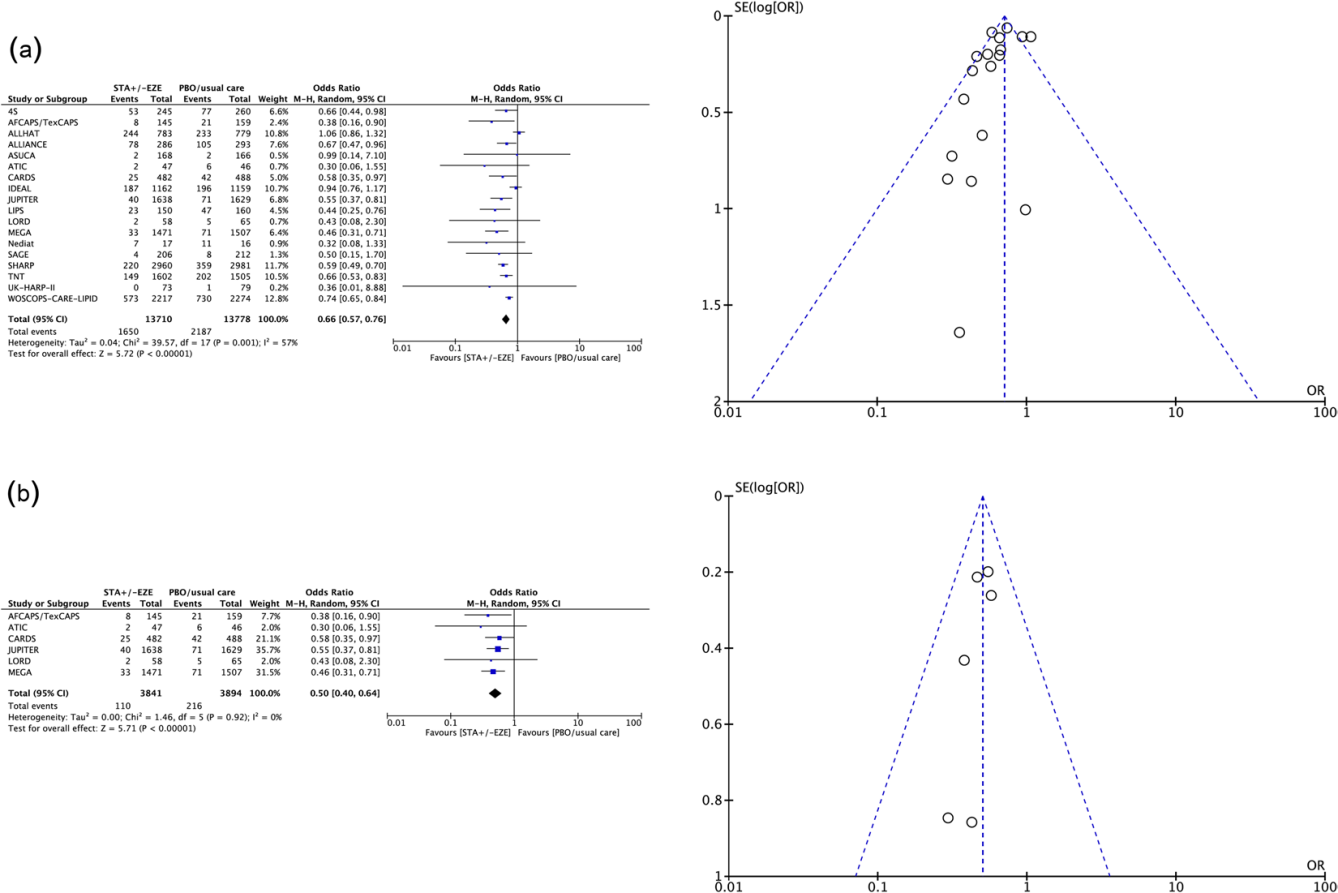
The effect of cholesterol-lowering treatment on MACEs when the eGFR is lower than 60 ml/min/1.73 m^2^ (excluding dialysis patients). (**a**) All trials. (**b**) Primary prevention trials. CI, confidence interval; eGFR, estimated glomerular filtration rate; EZE, ezetimibe; M-H, Mantel–Haenszel test; PBO, placebo; SE, standard error; STA, statins.

**Figure 3:**
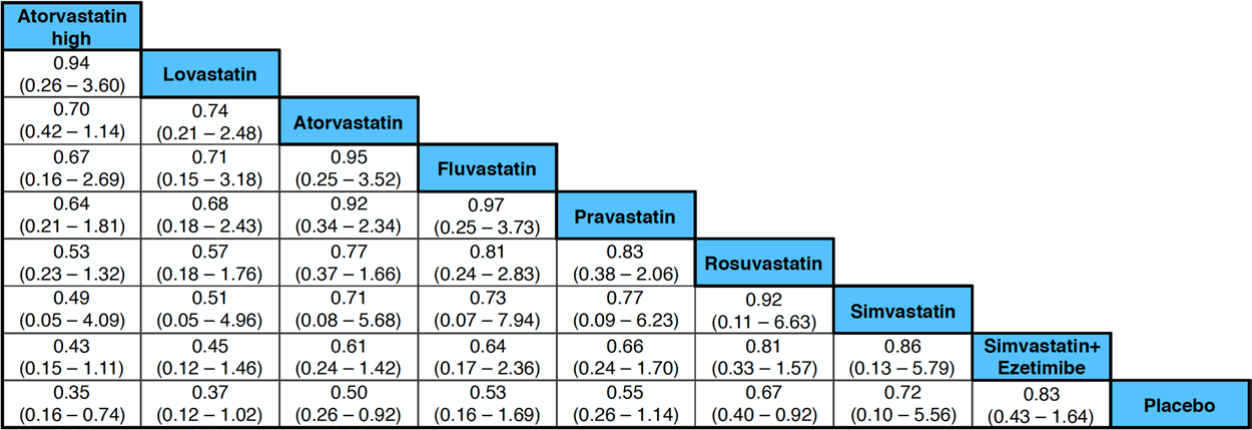
SUCRA-based ranking of all statins and the combination of simvastatin plus ezetimibe when the eGFR is lower than 60 ml/min/1.73 m^2^ (excluding dialysis patients). eGFR, estimated glomerular filtration rate; SUCRA, surface under the cumulative ranking.

**Figure 4:**
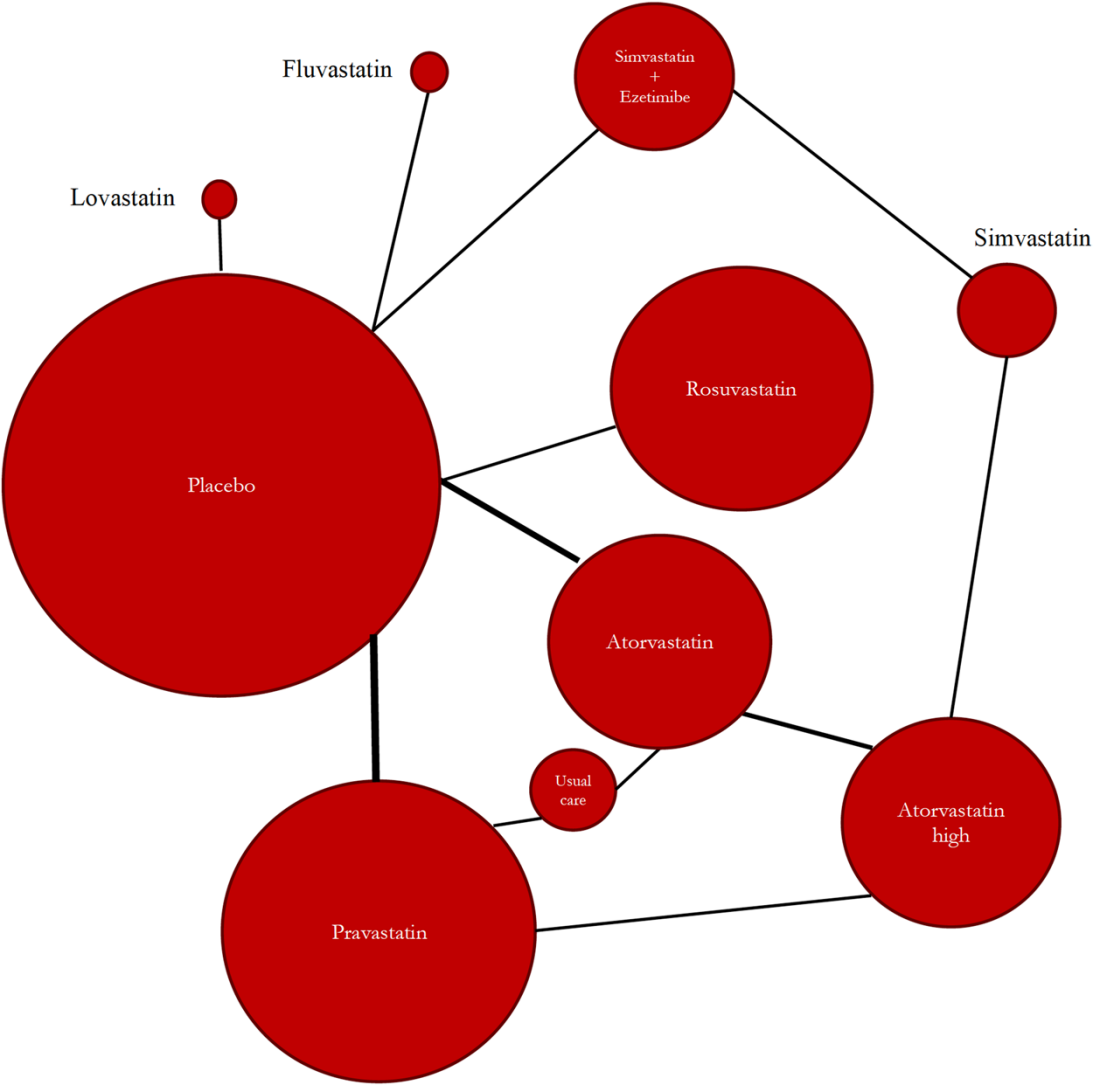
Network diagram for the competing cholesterol-lowering treatment strategies (Markov chain Monte Carlo simulation).

**Figure 5:**
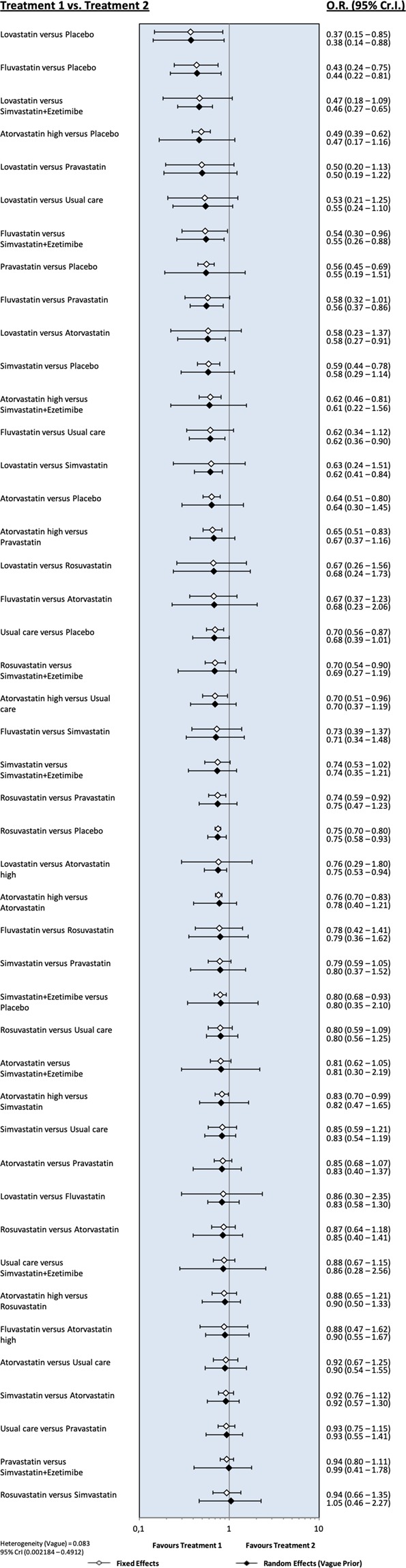
Network forest plot of the fixed and random effects of the competing cholesterol-lowering treatment strategies. CrI, credible intervals; OR, odds ratio.

**Figure 6:**
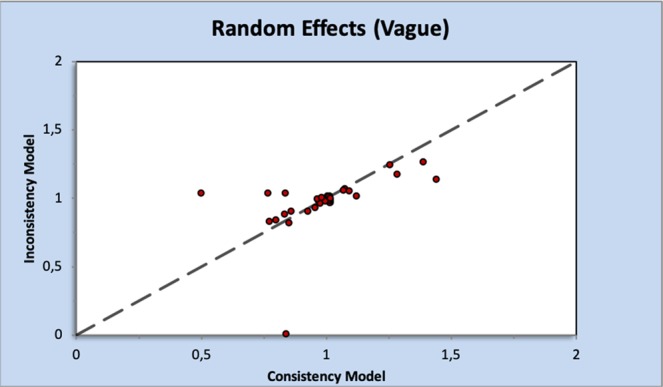
Inconsistency plot of the random effects for the competing cholesterol-lowering treatment strategies.

Moderate- to high-quality trials (Table S2) indicate that atorvastatin 80 mg daily, simvastatin 20 mg daily combined with ezetimibe 10 mg daily, simvastatin 20 mg daily and milligram-equivalent doses of fluvastatin, atorvastatin, rosuvastatin, pravastatin and lovastatin all reduced the incidence of major adverse cardiovascular events (MACEs) in patients with CKD KDIGO GFR categories G3a–G5 (excluding dialysis patients)^16–35^. Figure 2a,b shows the pooled odds ratios (OR) and 95% confidence intervals (95% CI) corresponding to pairwise meta-analysis of all trials conducted in this population (primary/secondary prevention; 0.66, 0.57 to 0.76) and those that enrolled only patients without known CVD (primary prevention; 0.50, 0.40 to 0.64). The heterogeneity (I^2^) was 57% and 0%, respectively. However, Bayesian network meta-analysis confirmed that compared to placebo, only atorvastatin 80 mg daily and atorvastatin and rosuvastatin at doses equivalent to simvastatin 20 mg daily reduced the odds of incident MACEs when the eGFR was lower than 60 ml/min/1.73 m^2^ (excluding dialysis patients). The ORs and 95% CIs for the abovementioned treatments were 0.35, 0.16 to 0.74; 0.50, 0.26 to 0.92; and 0.67, 0.40 to 0.92, respectively. Figure 3 shows a league table ranking all statins and the simvastatin/ezetimibe combination on the basis of the surface under the cumulative ranking (SUCRA)^40^. In the generated network diagram (Fig. 4), the heterogeneity (vague) was 0.08 (Fig. 5), and there was no relevant inconsistency (Fig. 6).

In persons with NKF/CKD KDIGO GFR categories G1–G2^17,22–35^ and in dialysis patients^13–18^, the ORs and 95% CIs for MACEs were 0.78, 0.71 to 0.85 and 0.95, 0.81 to 1.07, respectively (Figs S1 and S2).

### Treatment objectives evaluation

Serum levels of LDLc and/or CRP decreased in response to treatment with simvastatin 20 mg daily combined with ezetimibe 10 mg daily^17^, lovastatin 40 mg daily^37^, atorvastatin 10 mg daily^38^, and rosuvastatin 20 mg daily^39^, regardless of eGFR and history of diabetes or CVD. Most patients with early CKD (eGFR ≥ 60 ml/min/1.73 m^2^) had high LDLc levels before treatment^17,37–39^. A reduction in CRP of more than 50% was not observed. Reductions in LDLc and/or CRP of less than 50% were noted in patients with CKD in any KDIGO GFR category^37–39^.

Pairwise meta-analysis showed that, regardless of eGFR and excluding dialysis patients, reductions in both LDLc and CRP of less than 50%, and reductions in LDLc (</≥50%) and CRP (<50%) separately were associated with a low frequency of MACEs (Figs S3–S6)^17,37–39^. However, in the network meta-analysis, only the combined reduction in LDLc and CRP appeared to have the most pronounced impact on this outcome^37–39^. The SUCRA was 0.77 (Table 1)^40^. In the network diagram built by treatment objectives (Fig. S7), the heterogeneity (vague) was 0.14 (Fig. S8) and there was no inconsistency (Fig. S9). Meta-regression with the combined reduction in LDLc and CRP and cholesterol-lowering treatments was not possible given the number of studies included^41^.

**Table 1:** The ranking of the LDLc and CRP treatment objectives based on SUCRA.

Treatment objectives^†^	SUCRA^‡^
LDLc reduction < 50% plus CRP reduction < 50%	**0**.**7719**
LDLc reduction ≥ 50%	**0**.**6261**
LDLc reduction < 50%	**0**.**5933**
CRP reduction < 50%	**0**.**4995**
None	**0**.**0092**

^†^In a Bayesian context (Markov chain Monte Carlo simulation), the LDLc and/or CRP treatment objectives were the parameters used for ranking according to probabilities for being the best, the second best, the third best, and so on $P(o=b)$, $b=1,\ldots ,a$. ^‡^SUCRA for each treatment objective $o$ out of the $a$ competing treatment objectives requires calculation of the $a$ vector of the cumulative probabilities ${cum}_{o,b}$ to be among the $b$ best treatment objectives, $b=1,\ldots ,a$. Abbreviations: CRP, C-reactive protein; LDL-c; low-density lipoprotein cholesterol; SUCRA, surface under the cumulative ranking.

Although clear details were not obtained, post hoc analyses evaluating the combined reduction in LDLc and CRP as predictors of future MACEs in CKD patients treated with a statin or statin/ezetimibe combination were conducted following a retrospective biomarker-stratified design that allowed for the evaluation of codependent health technologies (Table S3)^37–39^.

## Discussion

### Key messages

Statins at doses equivalent to simvastatin 20 mg daily (even considering the addition of ezetimibe) and statins at higher doses showed similar efficacy in reducing MACEs when the eGFR was lower than 60 ml/min/1.73 m^2^: Only atorvastatin and rosuvastatin were associated with a clear benefit in terms of MACE reduction. Irrespective of eGFR, MACEs were less frequent in patients with a moderate reduction in LDLc and CRP than in patients with reductions in either of these two parameters separately.

LDLc and/or CRP decrease as a result of treatment with statins alone or in combination with ezetimibe in CKD patients^17,37–39^. However, it should not be forgotten that malnutrition and chronic inflammation appear quickly once the eGFR is lower than 60 ml/min/1.73 m^2^ ^9^. As a consequence, the levels of LDLc and other lipoproteins decrease, resulting in an increased risk of all-cause and CVD mortality^42^. The evaluation of the benefits of statins with or without ezetimibe along the wide spectrum of CKD is thus a real necessity.

This study found that most patients with early-stage CKD (eGFR ≥ 60 ml/min/1.73 m^2^) presented with high LDLc levels before treatment^17,37–39^ and that the reductions in LDLc and CRP were moderate regardless of the eGFR of the patients^37–39^; it is likely that in the early stages of CKD, patients develop high LDLc levels, which then decrease as a result of the treatment (similar to non-CKD patients). Importantly, CRP is more difficult to decrease in CKD patients, and this becomes even more challenging as the need for dialysis approaches^43^: Our meta-analytic calculations revealed that reductions in LDLc and CRP, compared to the sole reduction in LDLc, were associated with a low frequency of MACEs. In our opinion, both LDLc and CRP should be considered for CKD patients initiating cholesterol-lowering treatment, as is currently the case for LDLc for non-CKD patients.

This study explored which cholesterol-lowering treatment strategies are the best for treating CVD risk in this particular population. CKD constitutes the common final manifestation of a constellation of pathologies that affect kidneys in a chronic and irreversible way, so therapies should be adapted to the complex and intricate pathophysiology resulting from the incremental disease burden; evidence-based treatment strategies should thus be conceived as a pragmatic option to improve decision-making for the daily management of patients^3^.

Statins, in addition to lowering LDLc levels, exert anti-inflammatory, antioxidant, anti-proliferative and immunomodulatory effects, such as influencing plaque stability, normalizing sympathetic outflow, and preventing platelet aggregation, among other pleiotropic beneficial effects^44–46^.

The effects of statins on CVD risk in CKD patients have been discussed in the most recent systematic reviews and meta-analyses^47–50^ and other previous evidence summaries^51–53^. Our results are in line with the findings of these studies. In particular, our findings in the Bayesian network meta-analysis are consistent with those obtained in The Cholesterol Treatment Trialists (CTT) Collaboration meta-analysis of individual participant data published in 2016, which concluded that the efficacy of statins is modest in CKD^47^. However, our study cannot confirm the effect on cerebrovascular events that was presented in a pairwise meta-analysis conducted in 2015^48^, probably because this outcome was included as a composite of MACEs in the trials that were eligible for our meta-analysis. Our study also found that no effect of ezetimibe alone can be expected in individuals with CKD, and this finding is also consistent with the available evidence^54^.

### Strengths and limitations

To our knowledge, this study is the first to present a summary of the efficacy of statins with or without ezetimibe in relation to reductions in LDLc and CRP levels along the wide spectrum of CKD. Importantly, monitoring LDLc levels is no longer recommended by the latest guidelines from the KDIGO lipid management work group, and it should be reserved for instances in which the results would alter the management of patient treatment^10^. However, the KDIGO lipid management work group recommends a full lipid profile upon first presentation (a statement made on the basis of low-quality evidence)^10^; according to the findings of this meta-analysis, CRP should be measured in addition to LDLc before initiating cholesterol-lowering treatment in CKD patients.

Our approach guarantees the provision of evidence that pertinently answers the formulated review questions. The evidence was obtained from general sources to focused sources according to a method that resembles the “mixed-criteria” quality appraisal method of Wortman^55^. In addition, a multidisciplinary supervision mechanism was planned for contextualizing the search findings^56^. Nevertheless, some limitations must be mentioned. First, only 3 post hoc analyses of RCTs including patients with and without CKD provided evidence for the combined reduction in LDLc and CRP in CKD patients^37–39^. The criticism against attributing more value to the outcome of an unplanned analysis is difficult to ignore^57^. In addition, considering that there was no possibility to perform meta-regression^41^, our results should be considered prudently, particularly considering the current paucity of clinical research on CVD in CKD^3^, which should highlight the need for more research^58^. Second, publication bias is likely to affect our results. No unpublished studies were found, but asymmetry in the funnel plots may be a discouraging finding. Reporting bias occurs because significant results suggesting a beneficial effect of interventions are more likely to be published than nonsignificant results^59^. Caution is thus necessary when interpreting the manuscript, and common sense is required when applying our results into everyday clinical practice. Finally, the power of the trials evaluating lovastatin and fluvastatin was probably insufficient, resulting in an inappropriate ranking of all statins in the network meta-analysis^23,29^. Once more, caution is needed. For inflated associations, being fair with the interpretation of the results is an important consideration^60^.

### Conclusion and study contribution

In conclusion, statins with and without ezetimibe have a modest efficacy for reducing CVD risk in patients with CKD and eGFRs lower than 60 ml/min/1.73 m^2^. Irrespective of eGFR, if LDLc and CRP levels are high before treatment, reductions in such parameters are appropriate to obtain a benefit from cholesterol-lowering treatment. In other words, the use of statins should be initiated when levels of LDLc and CRP are high to reduce the CVD risk of patients. Importantly, patients with advanced CKD may present with low levels of LDLc. Such patients have a higher risk of CVD events, much like the risk of patients presenting with high levels of LDLc^42^. The benefit of cholesterol-lowering treatment in CKD patients is evident, but the impact on those with advanced CKD remains unclear. Finally, in accordance with the current guidelines, after an initial measurement, monitoring levels of LDLc is probably not required for CKD patients^10^. However, CRP should be assessed in addition to LDLc before initiating cholesterol-lowering treatment. Evidence-based treatment strategies may lead to individualized therapies for CKD patients.

## Methods

### Study design, eligibility criteria and systematic searches

These multistage pairwise and network meta-analyses were carried out and reported according to the PRISMA guidelines^56^. Two protocols were registered in the International Prospective Register of Systematic Reviews (PROSPERO) under the following registration IDs: CRD42017075166 and CRD42017055787. A two-stage systematic review design constitutes the basis of our multistage approach^58,61^. A systematic mapping (cholesterol-lowering treatment efficacy evaluation phase) followed by an in-depth systematic review (treatment objectives evaluation phase) was planned, and the former was complemented by an independent parallel one-stage systematic review (Material S1). An expert advisory group was formed to contextualize the search findings (E.A., F.J.A., J.B.-M. and M.V.).

The review questions corresponding to each of the 2 systematic reviews and the participants/population, intervention(s), and comparators are presented in Table 2. The primary outcome was incident MACEs, which included all fatal and nonfatal coronary events, including revascularization procedures, and all cerebrovascular events, including transient ischemic attacks (TIAs). The secondary outcome was material codependency when combining technologies related to treatment and the potential treatment effect modifiers for a low frequency of MACEs. The eligible studies were all randomized controlled trials (RCTs) with extended follow-up periods and post hoc analysis that were carried out on or that included CKD patients.

**Table 2:** Review questions and study eligibility.

	Systematic mapping (stage 1)/ systematic review support	In-depth meta-analysis (stage 2)
Review question	Are statins with or without ezetimibe efficacious in reducing CVD risk in patients with CKD?^§^	Are serum levels of LDLc and CRP related to CVD events in patients with CKD receiving treatment with statins alone or in combination with ezetimibe?
Participants/population	Adult individuals with NKF/CKD KDIGO GFR categories G1–G2, patients with CKD KDIGO GFR categories G3a–G5,^#^ and patients treated with chronic dialysis or kidney transplantation.^§^	Adult individuals with NKF/CKD KDIGO GFR categories G1–G2, patients with CKD KDIGO GFR categories G3a–G5, and patients treated with chronic dialysis or kidney transplantation.
Intervention(s)/exposures(s)	Statins with or without ezetimibe.^§^	Serum levels of LDLc and/or CRP in patients treated with statins with or without ezetimibe.
Comparators	Placebo/usual care.^@^	Serum levels of LDLc and/or CRP under placebo/usual care.^@^

^§^Only statins were studied, and patients treated with chronic dialysis or kidney transplantation were included in the systematic review to support systematic mapping. ^#^Individuals with CKD were divided according to eGFR (in ml/min/1.73 m^2^): ≥60 (KDIGO GFR categories G1–G2) or < 60 (G3a–G5). ^@^Usual care may include a statin if a statin at a higher dose or a statin/ezetimibe combination was already the intervention. Abbreviations: CKD, chronic kidney disease; CRP, C-reactive protein; CVD, cardiovascular disease; eGFR, estimated glomerular filtration rate; KDIGO, the Kidney Disease: Improving Global Outcomes; LDLc, low-density lipoprotein cholesterol; NKF, normal kidney function.

In parallel, published and unpublished literature sources were searched at Pharmacological Big Data Laboratory, Pharmacology and Therapeutics, Faculty of Medicine, University of Valladolid, Valladolid, Spain and Centro de Investigación en Salud Pública, Instituto de Investigación de la Facultad de Medicina Humana, Universidad de San Martín de Porres, Lima, Perú through September 2018 according to the homogenous PICO element-based search strategies that are available online from the systematic review protocols registered in PROSPERO (Material S2).

### Data collection and analyses

Anonymized datasets describing characteristics of the studies and their participants, interventions, comparators, outcomes, and follow-up periods were constructed. Before analysis, the risk of bias in the eligible studies was assessed using the standard tool produced by the Cochrane Collaboration^62^. A two-stage pairwise and network meta-analysis of aggregate-level data was planned (C.O.-S. and F.H.-G.). However, if there were not enough data or if there was a doubt about the comparability of the data, a two-staged systematic narrative synthesis was envisaged^63^.

The overall odds ratios and their 95% confidence intervals for the outcome of incident MACEs corresponding to cholesterol-lowering treatment (stage 1) and the treatment objectives of LDLc and/or CRP (stage 2) were obtained (Mantel-Haenszel random-effect method) through conventional pairwise meta-analysis. Heterogeneity was examined by computing the I² statistic (inconsistency) and the χ² statistic, and the presence of reporting bias was assessed by visual inspection of funnel plots of the estimates against their standard errors. A calculation of the regression coefficient corresponding to cholesterol-lowering treatment and the treatment objectives of LDLc and/or CRP (stage 2) was planned (random-effects meta-regression). Review Manager software (RevMan) version 5.3 (Cochrane Collaboration) and the ‘metareg’ macro from Stata version 12.1 (StataCorp) were used to conduct the pairwise meta-analytic calculations and meta-regression, respectively. Pooled ORs and the corresponding 95% credible intervals for incident MACEs (considered a ‘bad’ outcome) were calculated (random-effects model using vague priors with correction for zero values) via Bayesian network meta-analysis (Markov chain Monte Carlo simulation); the competing strategies (stage 1) and treatment objectives of LDLc and/or CRP (stage 2) were ranked based on SUCRA (surface under the cumulative ranking) after verifying convergence (Brooks-Gelman-Rubin method) and inconsistency. Network meta-analysis was performed using NetMetaXL software (Canadian Agency for Drugs and Technologies in Health and Cornerstone Research Group)^64^. Analysis of subgroups was planned at stage 1 (NKF/CKD KDIGO GFR categories G1–G2, CKD KDIGO GFR categories G3a–G5, chronic dialysis, transplantation) and stage 2 (LDLc reduction < 50% combined with CRP reduction < 50%, LDLc reduction < 50%, LDLc reduction ≥ 50%, CRP reduction < 50%).

Codependency when combining technologies related to the treatment and the potential treatment effect modifiers for a low frequency of MACEs (LDLc combined with CRP) was assessed using an adaptation of Merlin’s tool included in the guidelines for preparing a submission to the Pharmaceutical Benefits Advisory Committee (PBAC) from the Department of Health of Australia (F.H.-G. and A.M.-B.-M.)^65^. The tool sections of the economic evaluation and use of the medicine in practice were not considered.

## Supplementary information

Supplementary information file

## Data Availability

All data generated and analyzed during this study are included in this published article and the supplementary materials.
