# Supplementary material for: Cholesterol-Lowering Treatment in Chronic Kidney Disease: Multistage Pairwise and Network Meta-Analyses
Source: Sci Rep. 2019 Jun 20;9:8951. doi: 10.1038/s41598-019-45431-5 (PMC6586647; doi:10.1038/s41598-019-45431-5)
Supplement: Supplementary file 1 — Supplementary information file [file 41598_2019_45431_MOESM1_ESM.pdf]

Supplementary information for the following article:

Cholesterol-lowering treatment in chronic kidney disease: Multistage pairwise and network meta-analyses

Francisco Herrera-Gómez, M. Montserrat Chimeno, Débora Martín-García, Frank Lizaraso-Soto, Álvaro Maurtua-Briseño-Meiggs, Jesús Grande-Villoria, Juan Bustamante-Munguira, Eric Alamartine, Miquel Vilardell, Carlos Ochoa-Sangrador, F. Javier Álvarez

The Supplementary Materials for this manuscript include the following:

Material S1. Supplementary Methods.

Material S2. Links to registered systematic review protocols and search strategies.

Table S1. Participants, interventions, comparators, and outcomes in the eligible trials.

Table S2. Assessing risk of bias in the eligible trials.

Table S3. Evaluation of codependent health technologies.

Figure S1. The effect of cholesterol-lowering treatment on MACEs when the eGFR is 60 ml/min/1.73 m^2 or higher.

Figure S2. The effect of cholesterol-lowering treatment on MACEs in dialysis patients.

Figure S3. The effect of a combined reduction in LDLc and CRP of less than 50% regardless of eGFR.

Figure S4. The effect of a reduction in LDLc of less than 50% regardless of eGFR.

Figure S5. The effect of a reduction in LDLc of 50% or higher regardless of eGFR.

Figure S6. The effect of a reduction in CRP of less than 50% regardless of eGFR.

Figure S7. Network diagram of the LDLc and CRP treatment objectives regardless of eGFR and not including dialysis patients (Markov chain Monte Carlo simulation).

Figure S8. Network forest plot of fixed and random effects for the LDLc and CRP treatment objectives.

Figure S9. Inconsistency plot of the random effects for the LDLc and CRP treatment objectives.

Material S1

Supplementary Methods

According to our intention to produce a two-step summary, the cardiovascular protective efficacy of the strategies based on statins with or without ezetimibe in patients with chronic kidney disease (CKD) was assessed (cholesterol-lowering treatment efficacy evaluation phase); thereafter, low-density lipoprotein cholesterol (LDLc) and C-reactive protein (CRP) were evaluated as potential markers of major adverse cardiovascular events (MACE) in CKD patients who had received such treatment (treatment objectives evaluation phase). Consequently, a two-stage systematic review design was conceived. With the support of an independent parallel one-stage systematic review, systematic mapping (stage 1) allowed us to perform the cholesterol-lowering treatment efficacy evaluation phase. Subsequently, an in-depth systematic review (stage 2) allowed us to accomplish the treatment objectives evaluation phase.

At the systematic mapping stage, the eligible studies should have compared all strategies using statins alone or combined with ezetimibe and placebo or usual care (the use of a statin was considered usual care if the intervention was a statin at different doses than those planned for the statin used as a comparator or if the intervention was a statin/ezetimibe combination). At the in-depth systematic review stage, the included studies should have compared serum levels of LDLc and/or CRP between patients receiving the intervention and those assigned to the comparator.

MEDLINE via PubMed, Ovid and the Web of Science; EMBASE via Elsevier's Scopus; and the Cochrane Central Register of Controlled Trials (CENTRAL) were searched through September 2018. ClinicalTrials.gov, the EU Clinical Trials Register, and the United Kingdoms' ISRCTN registry were consulted to supplement the internet database searches. Gray literature sources were also scrutinized. DART-Europe E-Theses portal and Open Access Theses and Dissertations were searched to identify relevant PhD and master's theses. Manual searches in meeting abstract archives of the American Society of Nephrology (ASN) Kidney Week 2003 to 2017, the European Renal Association-European Dialysis and Transplant Association (ERA-EDTA) congress 2003 to 2018, and the International Society of Nephrology (ISN) World Congress of Nephrology 2001, 2003, 2005, 2007, 2009, 2011, 2013, 2015 and 2017 were performed to identify relevant abstracts. The reference lists of all eligible reports were scanned to identify all relevant studies cited by the included studies. The literature search was restricted to the English language.

Three different review teams, each with specific team conciliators, were formed to select studies (A.M.-B.-M., J.G.-V. and D.M.-G. for the systematic mapping; A.M.-B.-M., F.L.-S. and M.M.C. for the systematic review support of systematic mapping; and D.M.-G., F.H.-G. and M.M.C. for the in-depth systematic review): The screening of titles/abstracts and, subsequently, full-text report examination were carried out by two reviewers independently and disagreements that arose were solved with the participation of a third reviewer who acted as a conciliator. The corresponding authors of the included studies were contacted whenever possible to retrieve missing information and to confirm the study details.

Material S2

Links to registered systematic review protocols and search strategies

Two-stage systematic review

PROSPERO registration ID: CRD42017075166

**Systematic review protocol**<br><br>http://www.crd.york.ac.uk/PROSPERO/display_record.php?ID=CRD42017075166
**Search strategy**<br><br>https://www.crd.york.ac.uk/PROSPEROFILES/75166_STRATEGY_20171221.pdf

One-stage systematic review

PROSPERO registration ID: CRD42017055787

**Systematic review protocol**<br><br>http://www.crd.york.ac.uk/PROSPERO/display_record.php?ID=CRD42017055787
**Search strategy**<br><br>https://www.crd.york.ac.uk/PROSPEROFILES/55787_STRATEGY_20171221.pdf

Table S1. Participants, interventions, comparators, and outcomes in the eligible trials.

**Trial**<br>Detail	**Design**	**Follow-up** (yrs)	**Participants/population**<br>Characteristics	**Interventions**<br>(n)	**Comparators**<br>(n)	**Outcomes**<br>CVD-related/others	**Cointerventions**
**ALERT**[1,2]<br>International	RCT+EXT	5.1+2	≥65 years/male/DM/CVD (%):[€] 0.0/66.5/17.0/5.0<br>KDIGO GFR G3a–G5/LDLc <100 md/dl (%): 100.0/0.0<br>Causes of CKD (%): GN/AID (38.3), TIN/HTN (17.3), Unknown/other (16.1), ADPKD (15.3), DM (13.0).	Fluvastatin 40 mg/d (1050)	Placebo (1052)	MACE[&].	None
**4D**[3,4]<br>Germany	RCT+EXT	4+7.5	≥65 years/male/DM/CVD (%):[£] 68.5/54.0/100.0/47.2<br>KDIGO GFR G3a–G5/LDLc <100 md/dl (%): 100.0/23.7	Atorvastatin 20 mg/d (636)	Placebo (619)	MACE[&].<br>All-cause mortality.	None

			Causes of CKD (%): NA.				
**AURORA**[5] NCT00240331 International	RCT	3.2	≥65 years/male/DM/CVD (%):[£] 77.0/62.1/26.4/39.9 KDIGO GFR G3a–G5/LDLc <100 md/dl (%): 100.0/8.0 Causes of CKD (%): TIN/HTN (34.4), ADPKD/other (27.8), DM (19.3), GN/AID (18.5).	Rosuvastatin 10 mg/d (1391)	Placebo (1385)	MACE[&]. All-cause mortality, AVF stenosis/thrombosis.	None
**Nediat**[6] Sweden	RCT	3	≥65 years/male/DM/CVD (%):[$] 70.0/69.3/30.8/26.1 KDIGO GFR G3a–G5/LDLc <100 md/dl (%): 100.0/0.0 Causes of CKD (%): NA.	Atorvastatin 10 mg/d (70)	Placebo (73)	MACE[&]	None
**SHARP**[7]	RCT	4.9	≥65 years/male/DM/CVD	Simvastatin	Placebo (4620)	MACE[&].	None

NCT00125593<br>ISRCTN54137607<br>UK			(%):[$] 52.5/62.8/22.0/15.0<br>KDIGO GFR G3a–G5/LDLc<br><100 md/dl (%): 98.5/0.0<br>Causes of CKD (%):<br>TIN/HTN (29.0),<br>Unknown/other (28.0), GN<br>(17.0), DM (15.0), ADPKD<br>(11.0).	20 mg/d<br>plus ezetimibe<br>10 mg/d<br>(4650)			
**UK-HARP-II**[8]<br>UK	RCT	0.5	≥65 years/male/DM/CVD<br>(%):[$] 59.5/69.5/11.0/17.0<br>KDIGO GFR G3a–G5/LDLc<br><100 md/dl (%): 100.0/0.0<br>Causes of CKD (%):<br>Unknown/other (41.0),<br>TIN/HTN (26.0), GN/AID	Simvastatin<br>20 mg/d<br>plus ezetimibe<br>10 mg/d (102)	Simvastatin 20<br>mg/d (101)	MACE[&].<br>Safety and<br>tolerability.	None

			(14.0), ADPKD (13.5), DM (5.5).				
**ASUCA**[9] Japan	RCT	2	≥65 years/male/DM/CVD (%):[¥] 66.0/63.8/33.8/7.8 KDIGO GFR G3a–G5/LDLc <100 md/dl (%): 100.0/0.0 Causes of CKD (%): NA.	Atorvastatin 20 mg/d (176)	Placebo (173)	MACE[&]. eGFR decline.	Dietary and lifestyle counseling. RAAS inhibitor therapy.
**ATIC**[10] Netherlands	RCT	2	≥65 years/male/DM/CVD (%):[¥] 44.0/57.0/0.0/0.0 KDIGO GFR G3a–G5/LDLc <100 md/dl (%): 100.0/0.0 Causes of CKD (%): Unknown/other (43.0), HTN (37.0), ADPKD (20.0).	Pravastatin 40 mg/d (47)	Placebo (46)	MACE[&]. CC-IMT decrease, BA-FMD increase, eGFR decline, and reduction in UAE, oxLDL and plasma malondialdehyde.	RAAS inhibitor therapy. Vitamins B6, B12 and E.
**LORD**[11]	RCT	3	≥65 years/male/DM/CVD	Atorvastatin	Placebo (68)	MACE[&].	None

Northern Tasmania			(%):[¥] 76.0/65.0/10.0/0.0 KDIGO GFR G3a–G5/LDLc <100 md/dl (%): 100.0/0.0 Causes of CKD (%): ADPKD (44.2), GN (30.6), TIN/HTN (17.1), DM (8.1).	10 mg/d (64)		eGFR decline and reduction in UAE.	
**4S**[12] Scandinavian peninsula	PHA of RCT	5.5	≥65 years/male/DM/CVD (%):[¥] 43.0/81.4/4.5/15.4 KDIGO GFR G3a–G5/LDLc <100 md/dl (%): 11.4/0.0 Causes of CKD (%): NA.	Simvastatin 20 mg/d (1143)	Placebo (1171)	MACE[&].	None
**AFCAPS/ TexCAPS**[13] USA	PHA of RCT	5	≥65 years/male/DM/CVD (%):[¥] 22.0/87.9/2.3/0.0 KDIGO GFR G3a–G5/LDLc <100 md/dl (%): 4.6/0.0	Lovastatin 40 mg/d (3301)	Placebo (3304)	MACE[&].	None

			Causes of CKD (%): NA.				
**ALLHAT**[14] NCT00000542 USA	PHA of RCT	4.8	≥65 years/male/DM/CVD (%):[¥] 87.0/51.5/35.1/36.5 KDIGO GFR G3a–G5/LDLc <100 md/dl (%): 15.4/0.0 Causes of CKD (%): NA.	Pravastatin 40 mg/d (5085)	Usual care (5066)	MACE[&].	Nonstatin lipid-lowering medications (physician's judgement).
**ALLIANCE**[15] USA	PHA of RCT	6	≥65 years/male/DM/CVD (%):[¥] 80.0/82.2/22.1/64.5 KDIGO GFR G3a–G5/LDLc <100 md/dl (%): 23.7/0.0 Causes of CKD (%): NA.	Atorvastatin 10 mg/d (1217)	Usual care (1225)	MACE[&]. All-cause mortality.	Nonstatin lipid-lowering medications (physician's judgement).
**CARDS**[16] NCT00327418 UK	PHA of RCT	4	≥65 years/male/DM/CVD (%):[¥] 50.0/68.0/100.0/0.0 KDIGO GFR G3a–G5/LDLc <100 md/dl (%): 34.2/6.5	Atorvastatin 10 mg/d (1428)	Placebo (1410)	MACE[&].	None

			Causes of CKD (%): NA.				
**IDEAL**[17]<br>Norway	PHA of RCT	4.8	≥65 years/male/DM/CVD (%):¥ 80.8/60.0/12.0/51.3<br>KDIGO GFR G3a–G5/LDLc <100 md/dl (%): 26.2/5.0<br>Causes of CKD (%): NA.	Atorvastatin 80 mg/d (4439)	Simvastatin 20 mg/d (4449)	MACE&.<br>eGFR decline.	None
**JUPITER**[18]<br>NCT00239681<br>International	PHA of RCT	5	≥65 years/male/DM/CVD (%):¥ 82.3/61.8/0.0/0.0<br>KDIGO GFR G3a–G5/LDLc <100 md/dl (%): 18.0/5.0<br>Causes of CKD (%): NA.	Rosuvastatin 20 mg/d (8901)	Placebo (8901)	MACE&.	None
**LIPS**[19]<br>International	PHA of RCT	3.9	≥65 years/male/DM/CVD (%):¥ 94.0/84.1/12.2/100.0<br>KDIGO GFR G3a–G5/LDLc <100 md/dl (%): 19.9/4.0	Fluvastatin 40 mg/d (781)	Placebo (777)	MACE&.<br>Safety and tolerability.	Dietary and lifestyle counseling.

			Causes of CKD (%): NA.				
**MEGA**[20] NCT00211705 Japan	PHA of RCT	5.3	≥65 years/male/DM/CVD (%):¥ 60.0/23.1/19.8/0.0 KDIGO GFR G3a–G5/LDLc <100 md/dl (%): 41.3/0.0 Causes of CKD (%): Unknown/other (59.0), HTN (30.0), DM (11.0).	Pravastatin 20 mg/d (3533)	Placebo (3663)	MACE&. eGFR decline.	Dietary and lifestyle counseling.
**SAGE**[21] USA	PHA of RCT	1	≥65 years/male/DM/CVD (%):¥ 100.0/69.3/23.3/100.0 KDIGO GFR G3a–G5/LDLc <100 md/dl (%): 48.7/0.0 Causes of CKD (%): NA.	Atorvastatin 80 mg/d (433)	Pravastatin 40 mg/d (425)	MACE&. eGFR decline and safety and tolerability.	None
**TNT**[22]	PHA of	4.9	≥65 years/male/DM/CVD	Atorvastatin	Atorvastatin	MACE&.	None

NCT00327691<br>USA	RCT		(%):[¥] 38.2/81.1/14.8/100.0<br>KDIGO GFR G3a–G5/LDLc<br><100 md/dl (%): 32.2/10.0<br>Causes of CKD (%): NA.	80 mg/d<br>(4827)	10 mg/d<br>(4829)	eGFR decline.	
**WOSCOPS-CARE-LIPID**[23]<br>International	PHA of<br>RCTs		≥65 years/male/DM/CVD<br>(%):[¥] 42.5/89.1/7.2/54.8<br>KDIGO GFR G3a–G5/LDLc<br><100 md/dl (%): 26.7/0.0<br>Causes of CKD (%): NA.	Pravastatin<br>40 mg/d<br>(9338)	Placebo (9217)	MACE[&].<br>eGFR decline.	None
**PREVEND IT**[24,25]<br>Netherlands	RCT+EXT<br>2x2<br>factorial<br>design	4+5.5	≥65 years/male/DM/CVD<br>(%):[¥] 33.0/64.9/2.5/1.0<br>KDIGO GFR G3a–G5/LDLc<br><100 md/dl (%): 0.0/0.0<br>Causes of CKD (%): NA.	Pravastatin<br>40 mg/d (433)	Placebo (431)	MACE[&].	Fosinopril<br>20 mg/d

[€]All participants were patients who had undergone kidney transplantation [ALERT (the Assessment of LEscol in Renal Transplantation) Study]. [£]All participants were chronic dialysis patients (HD and PD) [studies: 4D (Die Deutche Diabetes Dialyse), AURORA (A study to evaluate the Use of Rosuvastatin in subjects On Regular haemodialysis: an Assessment of survival and cardiovascular events)]. [$]Participants were individuals with NKF/CKD KDIGO GFR categories G1–G2, patients with CKD KDIGO GFR categories G3a–G5, and patients treated with chronic dialysis [studies: Nediat, SHARP (Study of Heart And Renal Protection), UK-HARP-II (the second United Kingdom Heart and Renal Protection study)]. [¥]Participants were individuals with NKF/CKD KDIGO GFR categories G1–G2 and patients with CKD KDIGO GFR categories G3a–G5 but not dialysis patients [studies: ASUCA (ASsessment of clinical Usefulness in CKD patients with Atorvastatin), ATIC (the Antioxidant Therapy In Chronic renal insufficiency), LORD (the Lipid lowering and Onset of Renal Disease), 4S (the Scandinavian Simvastatin Survival Study), AFCAPS/TexCAPS (the Air Force/Texas Coronary Atherosclerosis Prevention Study), ALLHAT (the Antihypertensive and Lipid-Lowering treatment to prevent Heart Attack Trial (ALLHAT), ALLIANCE (the Aggressive Lipid-Lowering Initiation Abates New Cardiac Events), CARDS (the Collaborative AtoRvastatin in Diabetes Study), IDEAL (the Initiating Dialysis Early And Late), JUPITER (Justification for the Use of statins in Primary prevention–an Intervention Trial Evaluating Rosuvastatin), LIPS (Lescol Intervention Prevention Study), MEGA (Management of Elevated cholesterol in the primary prevention Group of Adult Japanese), SAGE (the Study Assessing Goals in the Elderly), TNT (Treating to New Targets), WOSCOPS (West of Scotland Coronary Prevention Study)-CARE (Cholesterol And Recurrent Events)-LIPID (Long-term Intervention with Pravastatin in Ischemic Disease (LIPID), PREVEND IT (the Prevention of REnal and Vascular ENdstage Disease Intervention Trial)]. [&]MACEs included all fatal and non-fatal coronary events including revascularization procedures and all cerebrovascular events including TIA. Abbreviations: ADPKD, autosomal-dominant polycystic kidney disease; AID, autoimmune disease; AVF, arteriovenous fistula; BA-FMD, brachial artery flow-mediated dilatation; CC-IMT, common carotid intima-media thickness; CKD, chronic kidney disease; DM, diabetes mellitus; eGFR, estimated glomerular filtration rate; ESKD, end-stage kidney disease; EXT, follow-up extension; GN,

glomerulonephritis; HD, hemodialysis; HTN, hypertension; KDIGO; Kidney Disease: Improving Global Outcomes; KTR, kidney transplant recipient; LDL-c; low-density lipoprotein cholesterol; MACE, major adverse cardiac event; NA, not available; NKF, normal kidney function; ox-LDL, plasma-oxidized low-density lipoprotein; PD, peritoneal dialysis; PHA, post hoc analysis; RAAS, renin-angiotensin-aldosterone system; RCT, randomized controlled trial; TIA, transient ischemic attack; TIN, tubulo-interstitial nephritis; UAE, urinary albumin excretion.

Table S2. Assessing risk of bias in the eligible trials.

Trials	Random sequence generation	Allocation concealment	Blinding of participants and personnel	Blinding of outcome assessment	Incomplete outcome data	Selective reporting	Other bias
**ALERT**	L	L	L	U	U	L	U
**4D**	L	L	L	L	U	L	U
**AURORA**	L	L	L	L	L	L	U
**Nediat**	L	L	U	U	U	L	U
**SHARP**	L	L	L	L	L	L	U
**UK-HARP-II**	L	L	U	U	U	L	U
**ASUCA**	L	L	U	U	U	L	U
**ATIC**	L	L	L	U	U	L	U
**LORD**	L	L	L	L	U	L	U
**4S**	L	L	U	U	U	U	U
**AFCAPS/TexCAPS**	L	L	U	U	U	U	U

**ALLHAT**	L	L	U	U	L	U	U
**ALLIANCE**	L	L	U	U	U	L	U
**CARDS**	L	L	U	U	L	U	U
**IDEAL**	L	L	U	U	U	U	U
**JUPITER**	L	L	U	U	L	U	U
**LIPS**	L	L	U	U	U	U	U
**MEGA**	L	L	U	U	L	U	U
**SAGE**	L	L	U	U	U	U	U
**TNT**	L	L	U	U	L	U	U
**WOSCOPS-CARE-LIPID**	L	L	U	U	U	U	U
**PREVEND IT**	L	L	L	U	U	L	U

The judgement for each of risk of bias domain is presented as (L), (U) or (H) to indicate low, unclear, or high risk of bias, respectively.

Table S3. Evaluation of codependent health technologies .

**Information requests**	**Comments**
**Section 1 – Context**	
**Details about the biomarker, the test and the medicine**	
1 (O) Current reimbursement arrangements.	The medicines and the test are available in developed countries, and the costs are affordable in most developing countries.
2 (T) Test sponsor.	Many sponsors are available.
3 (M) Medicine sponsor.	Many sponsors are available.
4 (O) Biomarker.	Serum levels of LDLc and CRP.
5 (T) Proposed test.	Determination of serum levels of LDLc and CRP.
6 (O) Medical condition or problem being managed.	CVD risk in patients with CKD.
7 (O) Clinical management pathways.	Treatment evaluation.
**Rationale for the codependency**	
8 (O) Definition of the biomarker.	Reduction in the levels of LDLc and CRP.
9 (O) Biological rationale for targeting that biomarker(s).	The risk of MACEs is lower when LDLc and CRP are reduced by treatment with the medicines.
10 (O) Other biomarker(s) to assess treatment effect of the medicine.	None.
11(O) Prevalence of the condition being	15%

targeted in the population that is likely to receive the test.	
**Proposed impact of codependent technologies on current clinical practice**	
12 (T) Consistency of the test results over time.	In the follow-up periods of the eligible studies, a low frequency of MACEs was observed when LDLc and CRP were reduced via treatment with the medicines.
13 (T) Use of the proposed test with other treatments and/or for other purposes.	NA
14 (T) Use of the test in the clinical management pathway.	The test is most likely to be an additional test to manage patients.
15 (T) Provision of the test.	The test is routinely used in hospitals of developed countries.
16 (T) Specimen or sample collection.	Peripheral blood.
17 (T) Use of the test for monitoring purposes (if relevant)	Treatment evaluation but not aiming for the titration of medicines.
18(O) Availability of other tests for the biomarker.	None.
**Section 2 – Clinical evaluation**	
**Direct evidence approach**	
**Section 2a Evidence of prognostic effect of the biomarker**	
19(O) Prognostic effect of the biomarker.	It can be assumed methodologically.
**Section 2d Clinical evaluation of the codependent technologies (combined)**	

20(O) Selection of the direct evidence.	Low-level direct evidence is available (retrospective biomarker-stratified trials).
21(O) Quality of the direct evidence.	The evidence is of adequate quality.

Item numbers are tagged with (T), (M) or (O), which indicate whether the item number is relevant to the test, the medicine or overlaps with both. Abbreviations: CKD, chronic kidney disease; CRP, C-reactive protein; CVD, cardiovascular disease; LDLc, low-density lipoprotein cholesterol; MACE, major adverse cardiovascular event.

Study or Subgroup	STA+/-EZE Events	STA+/-EZE Total	PBO/usual care Events	PBO/usual care Total	Weight	Odds Ratio M-H, Random, 95% CI
4S	173	892	249	909	8.1%	0.64 [0.51, 0.80]
AFCAPS/TexCAPS	108	3156	162	3145	7.1%	0.65 [0.51, 0.84]
ALLHAT	898	4302	964	4287	13.8%	0.91 [0.82, 1.01]
ALLIANCE	211	931	228	932	8.4%	0.90 [0.73, 1.12]
CARDS	58	946	85	922	4.6%	0.64 [0.45, 0.91]
IDEAL	342	3263	409	3279	11.2%	0.82 [0.71, 0.96]
JUPITER	102	7259	180	7269	7.3%	0.56 [0.44, 0.72]
LIPS	99	631	125	617	5.9%	0.73 [0.55, 0.98]
MEGA	65	2062	73	2156	4.7%	0.93 [0.66, 1.30]
PREVEND-IT	60	433	60	431	3.9%	0.99 [0.68, 1.46]
SAGE	1	227	2	213	0.1%	0.47 [0.04, 5.19]
SHARP	3	44	3	44	0.3%	1.00 [0.19, 5.25]
TNT	254	3225	307	3324	10.2%	0.84 [0.71, 1.00]
WOSCOPS-CARE-LIPID	1017	6159	1281	6174	14.4%	0.76 [0.69, 0.83]
**Total (95% CI)**		**33530**		**33702**	**100.0%**	**0.78 [0.71, 0.85]**
Total events	3391		4128			

Heterogeneity: $Tau^2 = 0.01$; $Chi^2 = 27.85$, df = 13 (P = 0.009); $I^2 = 53\%$
Test for overall effect: Z = 5.70 (P < 0.00001)

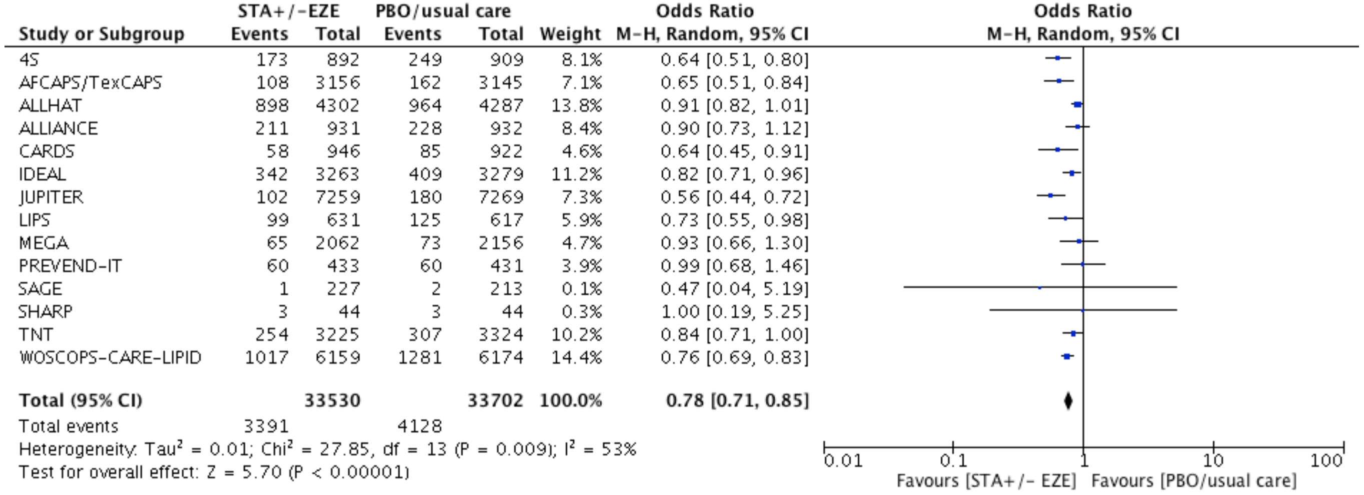

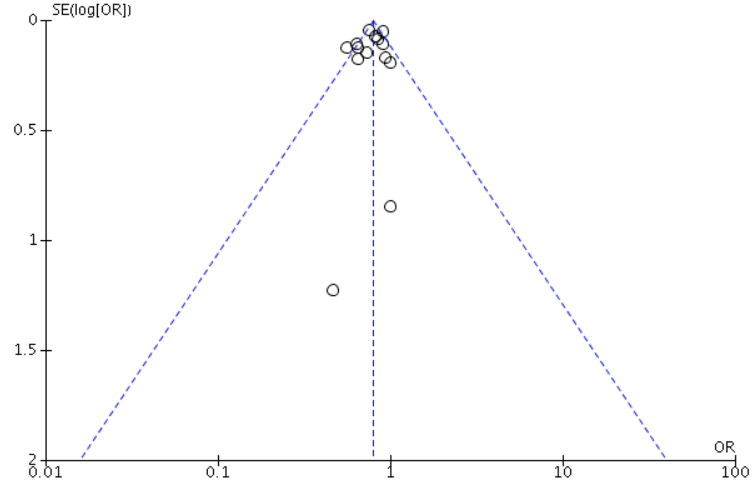

Figure S1. The effect of cholesterol-lowering treatment on MACEs when the eGFR is 60 ml/min/1.73 m^2 or higher. CI, confidence interval; eGFR, estimated glomerular filtration rate; EZE, ezetimibe; M-H, Mantel–Haenszel test; PBO, placebo; SE, standard error; STA, statins.

Study or Subgroup	STA+/-EZE Events	STA+/-EZE Total	PBO/usual care Events	PBO/usual care Total	Weight	Odds Ratio M-H, Random, 95% CI
4D	131	619	138	636	18.7%	0.97 [0.74, 1.27]
AURORA	324	1389	324	1384	44.0%	1.00 [0.83, 1.19]
Nediat	42	53	46	57	1.6%	0.91 [0.36, 2.32]
SHARP	230	1533	246	1490	35.5%	0.89 [0.73, 1.09]
UK-HARP-II	0	29	1	22	0.1%	0.24 [0.01, 6.26]
**Total (95% CI)**		**3623**		**3589**	**100.0%**	**0.95 [0.85, 1.07]**
Total events	727		755			

Heterogeneity: $Tau^2 = 0.00$; $Chi^2 = 1.36$, df = 4 (P = 0.85); $I^2 = 0\%$

Test for overall effect: Z = 0.87 (P = 0.39)

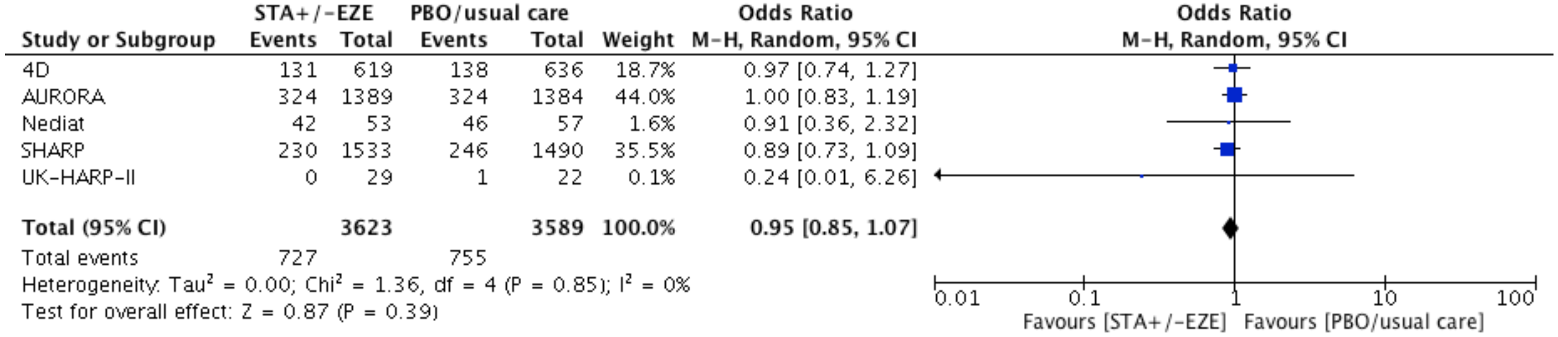

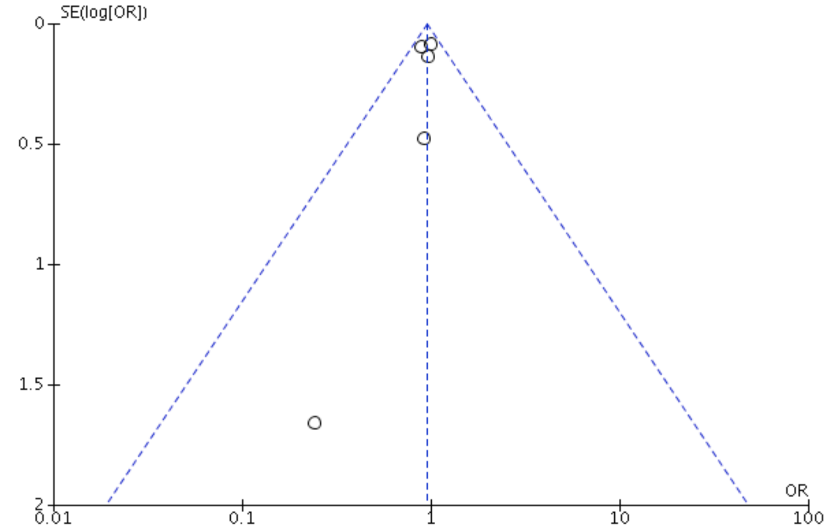

Figure S2. The effect of cholesterol-lowering treatment on MACEs in dialysis patients. CI, confidence interval; EZE, ezetimibe; M-H, Mantel–Haenszel test; PBO, placebo; SE, standard error; STA, statins.

Study or Subgroup	Treat obj Events	Treat obj Total	Non-treat obj Events	Non-treat obj Total	Weight	Odds Ratio M-H, Random, 95% CI
AFCAPS/TexCAPS	1	36	4	69	1.9%	0.46 [0.05, 4.32]
CARDS	9	119	39	377	16.5%	0.71 [0.33, 1.51]
JUPITER	41	2921	189	7832	81.6%	0.58 [0.41, 0.81]
**Total (95% CI)**		**3076**		**8278**	**100.0%**	**0.59 [0.44, 0.81]**
Total events	51		232			

Heterogeneity: $Tau^2 = 0.00$; $Chi^2 = 0.29$, df = 2 (P = 0.86); $I^2 = 0\%$

Test for overall effect: Z = 3.33 (P = 0.0009)

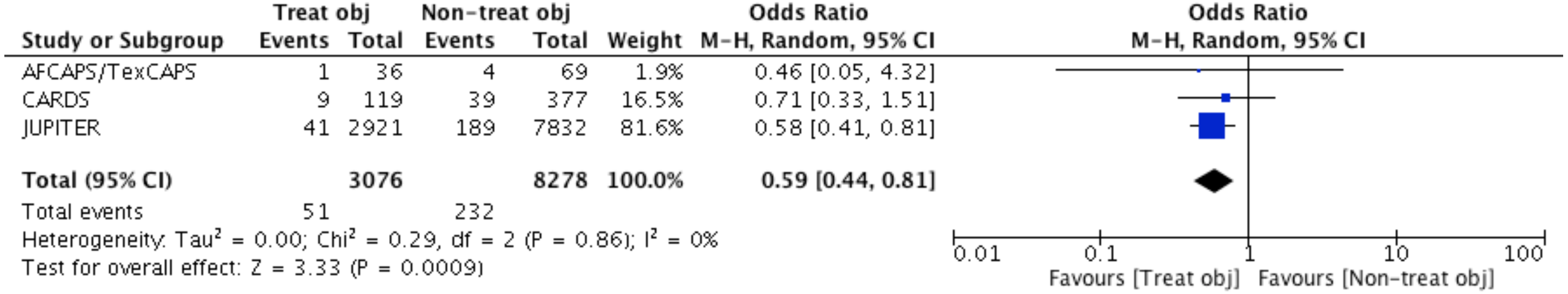

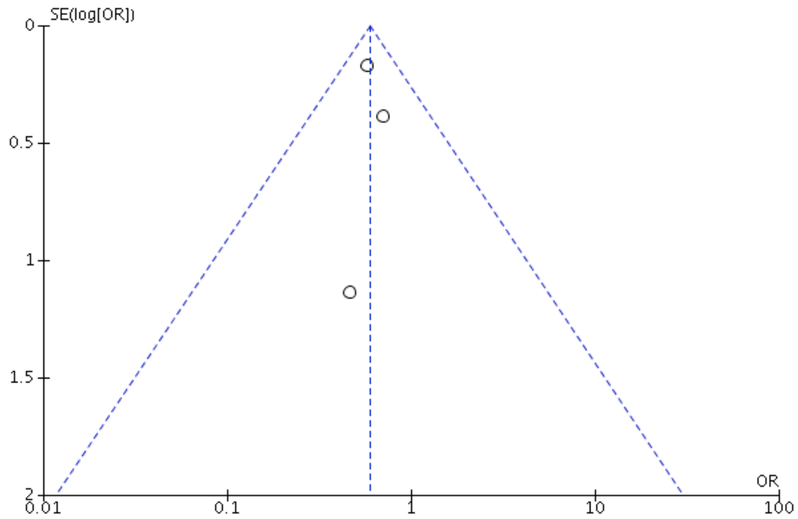

Figure S3. The effect of a combined reduction in LDLc and CRP of less than 50% regardless of eGFR. CI, confidence interval; CRP, C-reactive protein; eGFR, estimated glomerular filtration rate; LDLc, low-density lipoprotein cholesterol; M-H, Mantel–Haenszel test; SE, standard error.

Study or Subgroup	Treat obj Events	Treat obj Total	Non-treat obj Events	Non-treat obj Total	Weight	Odds Ratio M-H, Random, 95% CI
AFCAPS/TexCAPS	2	72	4	69	1.5%	0.46 [0.08, 2.62]
CARDS	15	243	39	377	12.1%	0.57 [0.31, 1.06]
JUPITER	7	636	34	1410	6.9%	0.45 [0.20, 1.02]
SHARP	135	1190	174	1130	79.5%	0.70 [0.55, 0.89]
**Total (95% CI)**		**2141**		**2986**	**100.0%**	**0.66 [0.53, 0.82]**
Total events	159		251			

Heterogeneity: $Tau^2 = 0.00$; $Chi^2 = 1.48$, df = 3 (P = 0.69); $I^2 = 0\%$
Test for overall effect: Z = 3.78 (P = 0.0002)

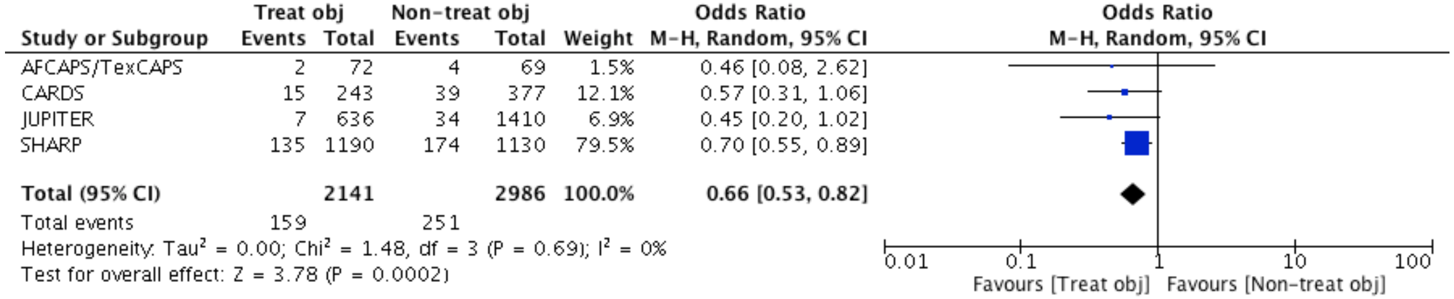

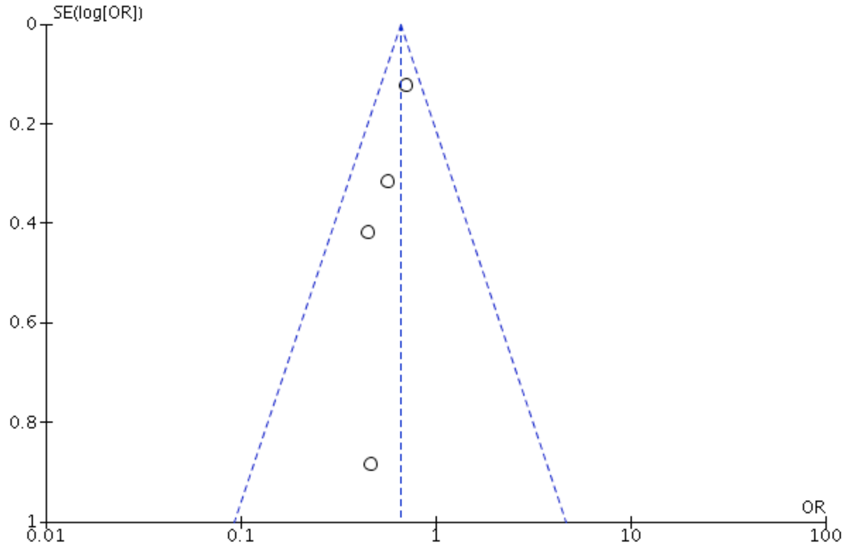

Figure S4. The effect of a reduction in LDLc of less than 50% regardless of eGFR. CI, confidence interval; eGFR, estimated glomerular filtration rate; LDLc, low-density lipoprotein cholesterol; M-H, Mantel–Haenszel test; SE, standard error.

Study or Subgroup	Treat obj Events	Treat obj Total	Non-treat obj Events	Non-treat obj Total	Weight	Odds Ratio M-H, Random, 95% CI
AFCAPS/TexCAPS	2	73	4	69	1.3%	0.46 [0.08, 2.58]
CARDS	10	143	39	377	7.5%	0.65 [0.32, 1.34]
JUPITER	12	753	34	1410	8.9%	0.66 [0.34, 1.27]
SHARP	202	1800	174	1130	82.3%	0.69 [0.56, 0.86]
**Total (95% CI)**		**2769**		**2986**	**100.0%**	**0.68 [0.56, 0.83]**
Total events	226		251			

Heterogeneity: $Tau^2 = 0.00$; $Chi^2 = 0.26$, df = 3 (P = 0.97); $I^2 = 0\%$

Test for overall effect: Z = 3.76 (P = 0.0002)

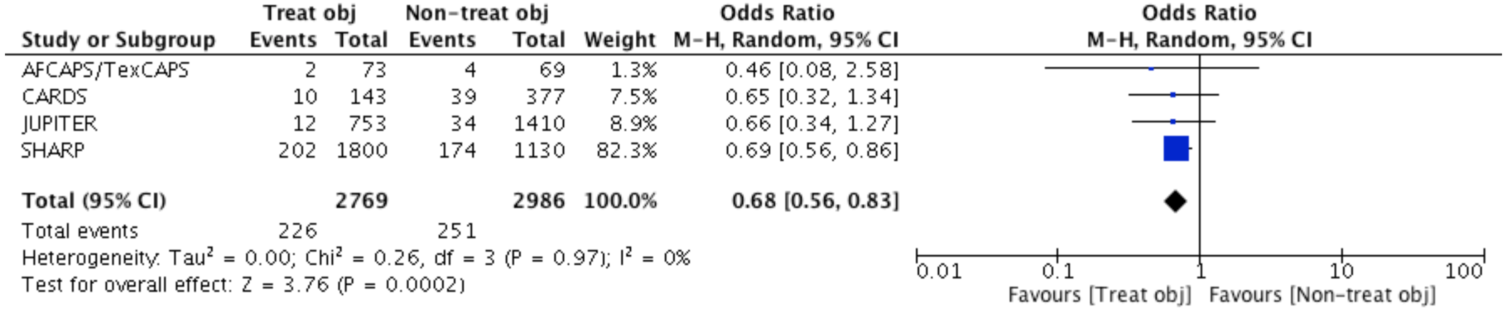

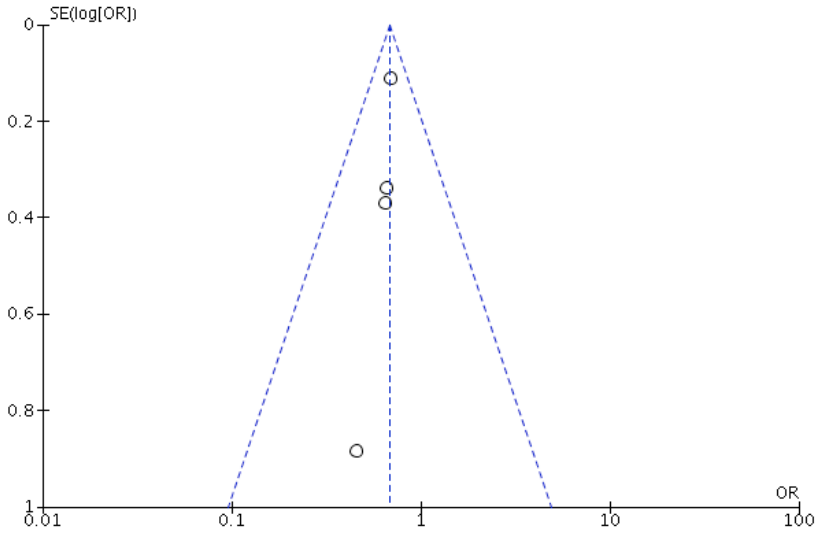

Figure S5. The effect of a reduction in LDLc of 50% or higher regardless of eGFR. CI, confidence interval; eGFR, estimated glomerular filtration rate; LDLc, low-density lipoprotein cholesterol; M-H, Mantel–Haenszel test; SE, standard error.

Study or Subgroup	Treat obj Events	Treat obj Total	Non-treat obj Events	Non-treat obj Total	Weight	Odds Ratio M-H, Random, 95% CI
CARDS	7	132	39	377	40.6%	0.49 [0.21, 1.11]
JUPITER	11	746	34	1410	59.4%	0.61 [0.31, 1.20]
**Total (95% CI)**		**878**		**1787**	**100.0%**	**0.55 [0.33, 0.94]**
Total events	18		73			

Heterogeneity: $Tau^2 = 0.00$; $Chi^2 = 0.16$, df = 1 (P = 0.69); $I^2 = 0\%$

Test for overall effect: Z = 2.19 (P = 0.03)

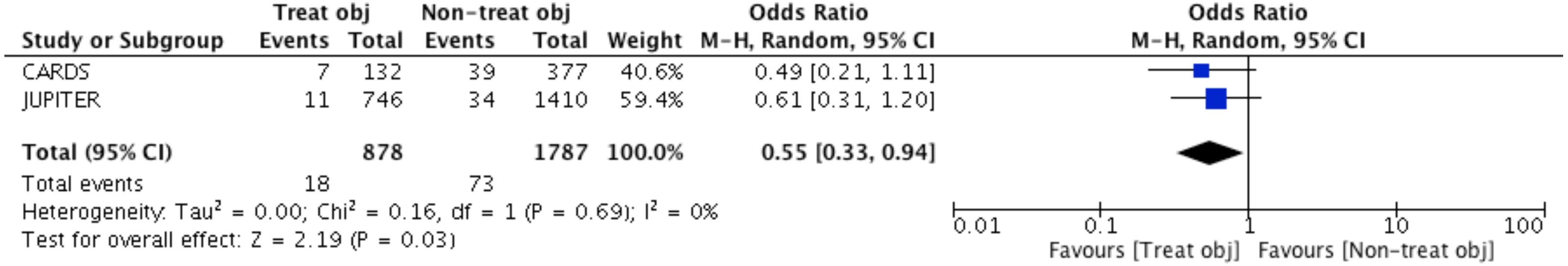

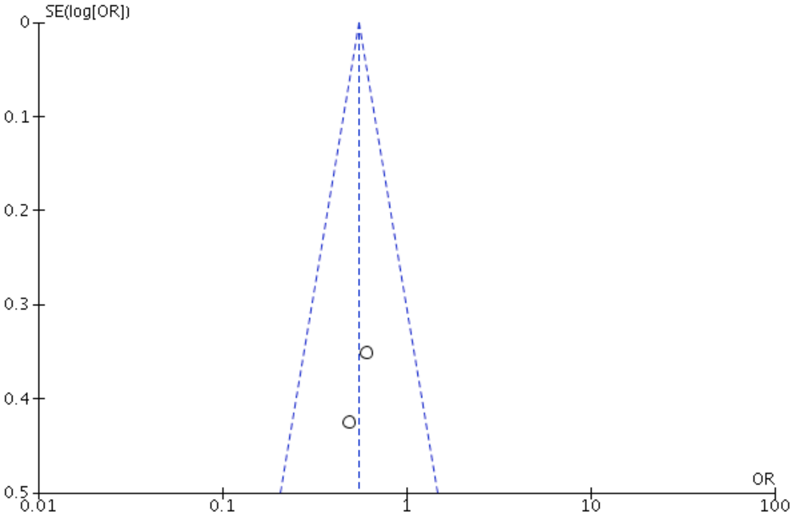

Figure S6. The effect of a reduction in CRP of less than 50% regardless of eGFR. CI, confidence interval; CRP, C-reactive protein; eGFR, estimated glomerular filtration rate; M-H, Mantel–Haenszel test; SE, standard error

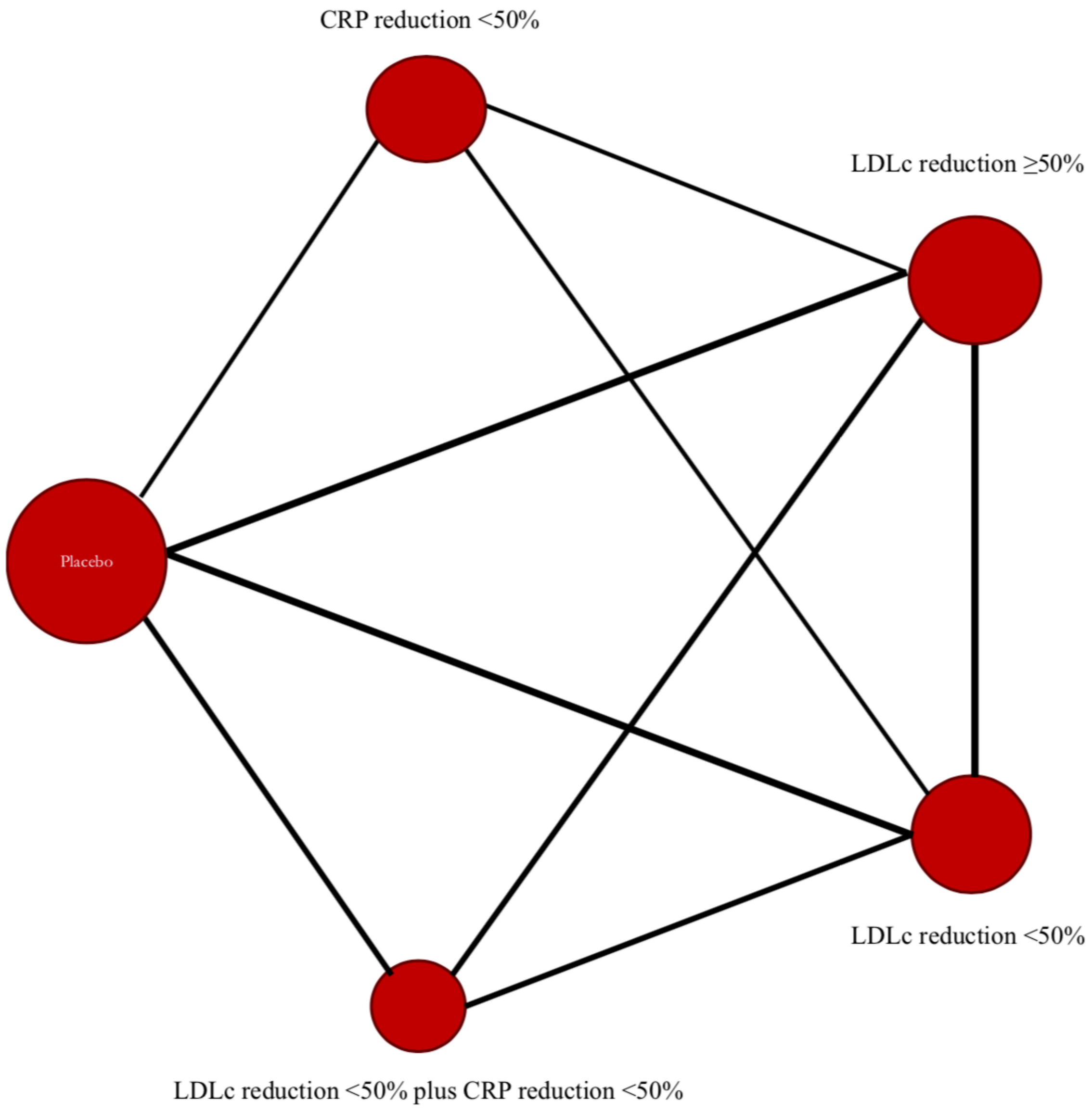

Figure S7. Network diagram of the LDLc and CRP treatment objectives regardless of eGFR and not including dialysis patients (Markov chain Monte Carlo simulation). CRP, C-reactive protein; LDLc, low-density lipoprotein cholesterol.

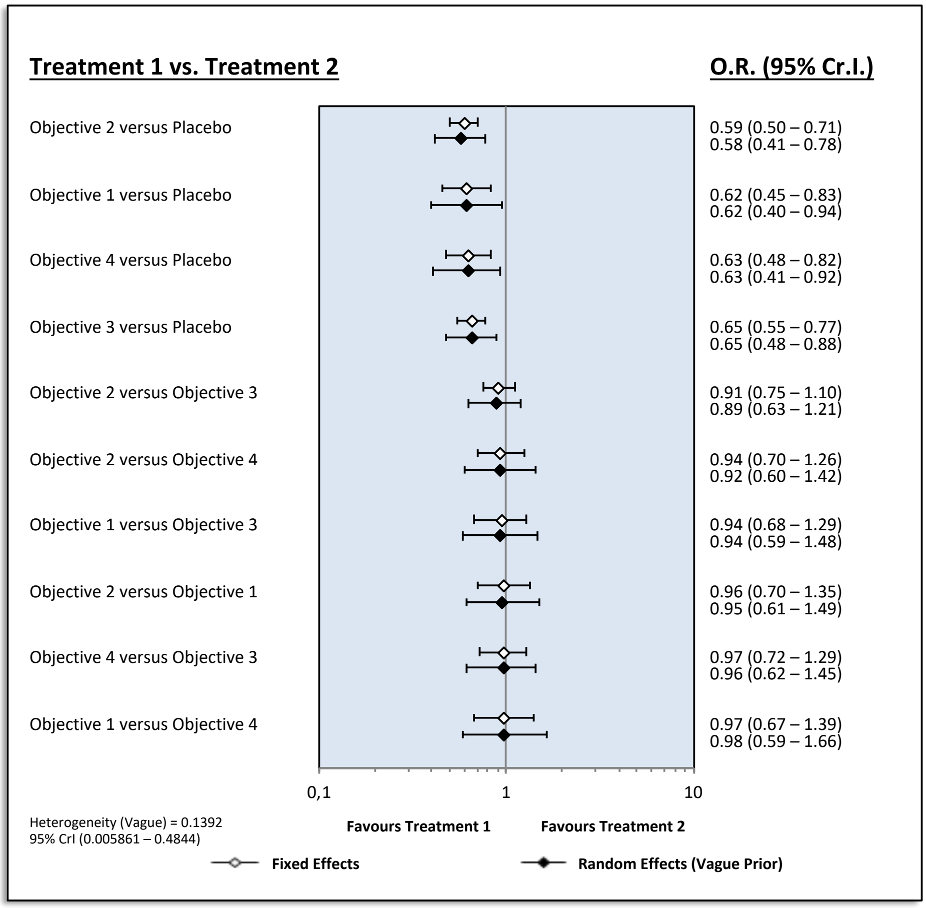

Figure S8. Network forest plot of fixed and random effects for the LDLc and CRP treatment objectives.

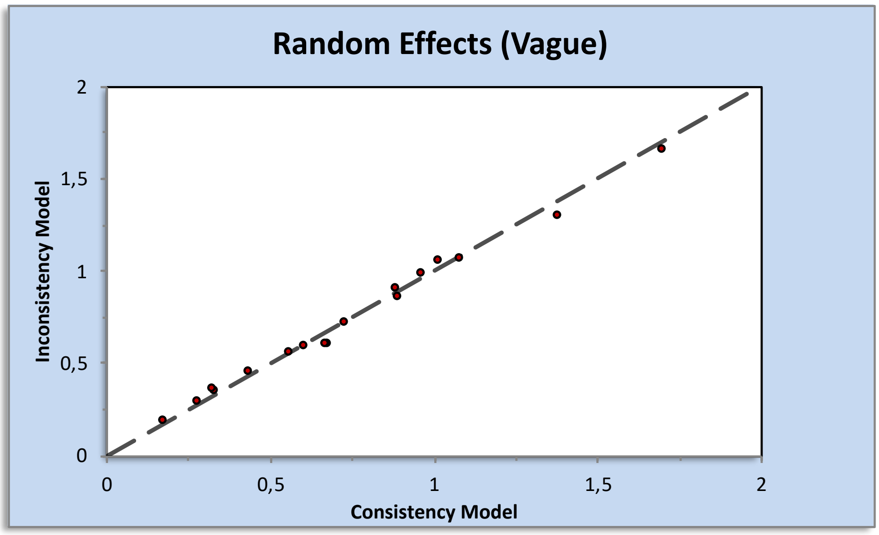

Figure S9. Inconsistency plot of the random effects for the LDLc and CRP treatment objectives.

References

1. Holdaas, H. *et al*. Effect of fluvastatin on cardiac outcomes in renal transplant recipients: a multicentre, randomised, placebo-controlled trial. *Lancet* **361**, 2024–2031 (2003). http://dx.doi.org/10.1016/S0140-6736(03)13638-0
2. Holdaas, H. *et al*. Long-term cardiac outcomes in renal transplant recipients receiving fluvastatin: the ALERT extension study. *Am. J. Transplant.* **5**, 2929–2936 (2005). http://dx.doi.org/10.1111/j.1600-6143.2005.01105.x
3. Wanner, C. *et al*. Atorvastatin in patients with type 2 diabetes mellitus undergoing hemodialysis. *N. Engl. J. Med.* **353**, 238–248 (2005). http://dx.doi.org/10.1056/NEJMoa043545
4. Krane, V. *et al*. Long-term effects following 4 years of randomized treatment with atorvastatin in patients with type 2 diabetes mellitus on hemodialysis. *Kidney Int.* **89**, 1380–1387 (2016). http://dx.doi.org/10.1016/j.kint.2015.12.033
5. Fellström, B. C. *et al*. Rosuvastatin and cardiovascular events in patients undergoing hemodialysis. *N. Engl. J. Med.* **360**, 1395–1407 (2009). http://dx.doi.org/10.1056/NEJMoa0810177
6. Stegmayr, B. G. *et al*. Low-dose atorvastatin in severe chronic kidney disease patients: a randomized, controlled endpoint study. *Scand. J. Urol. Nephrol.* **39**, 489–497 (2005). http://dx.doi.org/10.1080/00365590500329304
7. Baigent, C. *et al*. The effects of lowering LDL cholesterol with simvastatin plus ezetimibe in patients with chronic kidney disease (Study of Heart and Renal Protection): a randomised placebo-controlled trial. *Lancet* **377**, 2181–2192 (2011). http://dx.doi.org/10.1016/S0140-6736(11)60739-3

8. Landray, M. *et al*. The second United Kingdom Heart and Renal Protection (UK-HARP-II) Study: a randomized controlled study of the biochemical safety and efficacy of adding ezetimibe to simvastatin as initial therapy among patients with CKD. *Am. J. Kidney Dis.* **47**, 385–395 (2006). http://dx.doi.org/10.1053/j.ajkd.2005.11.018
9. Ueshima, K. *et al*. Effects of atorvastatin on renal function in patients with dyslipidemia and chronic kidney disease: rationale and design of the ASsessment of clinical Usefulness in CKD patients with Atorvastatin (ASUCA) trial. *Clin. Exp. Nephrol.* **17**, 211–217 (2013). http://dx.doi.org/10.1007/s10157-012-0676-5
10. Nanayakkara, P. W. *et al*. Effect of a treatment strategy consisting of pravastatin, vitamin E, and homocysteine lowering on carotid intima-media thickness, endothelial function, and renal function in patients with mild to moderate chronic kidney disease: results from the Anti-Oxidant Therapy in Chronic Renal Insufficiency (ATIC) Study. *Arch. Intern. Med.* **167**, 1262–1270 (2007). http://dx.doi.org/10.1001/archinte.167.12.1262
11. Fassett, R. G., Robertson, I. K., Ball, M. J., Geraghty, D. P., Coombes, J. S. Effect of atorvastatin on kidney function in chronic kidney disease: a randomised double-blind placebo-controlled trial. *Atherosclerosis* **213**, 218–224 (2010). http://dx.doi.org/10.1016/j.atherosclerosis.2010.07.053
12. Chonchol, M., Cook, T., Kjekshus, J., Pedersen, T. R., Lindenfeld, J. Simvastatin for secondary prevention of all-cause mortality and major coronary events in patients with mild chronic renal insufficiency. *Am. J. Kidney Dis.* **49**, 373–382 (2007). http://dx.doi.org/10.1053/j.ajkd.2006.11.043
13. Kendrick, J., Shlipak, M. G., Targher, G., Cook, T., Lindenfeld, J., Chonchol, M. Effect of lovastatin on primary prevention of cardiovascular events in mild CKD and kidney

function loss: a post hoc analysis of the Air Force/Texas Coronary Atherosclerosis Prevention Study. *Am. J. Kidney Dis.* **55**, 42–49 (2010). http://dx.doi.org/10.1053/j.ajkd.2009.09.020

14. Rahman, M. *et al*. Pravastatin and cardiovascular outcomes stratified by baseline eGFR in the lipid- lowering component of ALLHAT. *Clin. Nephrol.* 80, 235–248 (2013). http://dx.doi.org/10.5414/CN107922
15. Koren, M. J. *et al*. Focused atorvastatin therapy in managed-care patients with coronary heart disease and CKD. *Am. J. Kidney Dis.* **53**, 741–750 (2009). http://dx.doi.org/10.1053/j.ajkd.2008.11.025
16. Colhoun, H. M. *et al*. Effects of atorvastatin on kidney outcomes and cardiovascular disease in patients with diabetes: an analysis from the Collaborative Atorvastatin Diabetes Study (CARDS). *Am. J. Kidney Dis.* **54**, 810–819 (2009). http://dx.doi.org/10.1053/j.ajkd.2009.03.022
17. Holme, I. *et al*. Cardiovascular outcomes and their relationships to lipoprotein components in patients with and without chronic kidney disease: results from the IDEAL trial. *J. Intern. Med.* **267**, 567–575 (2010). http://dx.doi.org/10.1111/j.1365-2796.2009.02176.x
18. Ridker, P. M., MacFadyen, J., Cressman, M., Glynn, R. J. Efficacy of rosuvastatin among men and women with moderate chronic kidney disease and elevated high-sensitivity C-reactive protein: a secondary analysis from the JUPITER (Justification for the Use of Statins in Primary Prevention–an Intervention Trial Evaluating Rosuvastatin) trial. *J. Am. Coll. Cardiol.* **55**, 1266–1273 (2010). http://dx.doi.org/10.1016/j.jacc.2010.01.020

19. Lemos, P. A. *et al*. Long-term fluvastatin reduces the hazardous effect of renal impairment on four-year atherosclerotic outcomes (a LIPS substudy). *Am. J. Cardiol.* **95**, 445–451 (2005). http://dx.doi.org/10.1016/j.amjcard.2004.10.008
20. Nakamura, H. *et al*. Pravastatin and cardiovascular risk in moderate chronic kidney disease. *Atherosclerosis* **206**, 512–517 (2009). http://dx.doi.org/10.1016/j.atherosclerosis.2009.03.031
21. Deedwania, P. C., Stone, P. H., Fayyad, R. S., Laskey, R. E., Wilson, D. J. Improvement in Renal Function and Reduction in Serum Uric Acid with Intensive Statin Therapy in Older Patients: A Post Hoc Analysis of the SAGE Trial. *Drugs Aging.* **32**, 1055–1065 (2015). http://dx.doi.org/10.1007/s40266-015-0328-z
22. Shepherd, J. *et al*. Intensive lipid lowering with atorvastatin in patients with coronary heart disease and chronic kidney disease: the TNT (Treating to New Targets) study. *J. Am. Coll. Cardiol.* **51**, 1448–1454 (2008). http://dx.doi.org/10.1016/j.jacc.2007.11.072
23. Tonelli, M. *et al*. Effect of pravastatin on cardiovascular events in people with chronic kidney disease. *Circulation* **110**, 1557–1563 (2004). http://dx.doi.org/10.1161/01.CIR.0000143892.84582.60
24. Asselbergs, F. W. *et al*. Effects of fosinopril and pravastatin on cardiovascular events in subjects with microalbuminuria. *Circulation* **110**, 2809–2816 (2004). http://dx.doi.org/10.1161/01.CIR.0000146378.65439.7A
25. Brouwers, F. P. *et al*. Long-term effects of fosinopril and pravastatin on cardiovascular events in subjects with microalbuminuria: Ten years of follow-up of Prevention of Renal and Vascular End-stage Disease Intervention Trial (PREVEND IT). *Am. Heart J.* **161**, 1171–1178 (2011). http://dx.doi.org/10.1016/j.ahj.2011.03.028
